# Modeling the radiolysis in H_2_O : HCOOH ice: connecting laboratory experiments with astronomical measurements

**DOI:** 10.1039/d6ra03525f

**Published:** 2026-08-05

**Authors:** C. M. F. Fargnoli, L. Moraes, S. Pilling

**Affiliations:** a Universidade do Vale do Paraíba (UNIVAP) São José dos Campos SP 12244-000 Brazil carolinemilena499@gmail.com leonardomoraes580@gmail.com sergiopilling@yahoo.com.br

## Abstract

We present a computational study of radiolytic chemistry and desorption dynamics in astrophysical ice composed of H_2_O : HCOOH (1 : 1), irradiated with Ni^11+^ ions at 46 MeV and 15 K. Simulations were performed using the PROCODA model, calibrated with experimental data provided by GANIL in France in 2014, to investigate molecular evolution and desorption processes with temporal resolution. The results reveal a rapid progression towards chemical equilibrium at approximately 2000 s of irradiation, followed by the stabilization of major species such as CO, CO_2_, CH_3_OH, H_2_CO, OH, and HCO. The model also predicts dynamic and time-dependent desorption profiles, with transient peaks reflecting the evolution of the physicochemical environment within the ice. The simulated molecular abundances in equilibrium show some agreement with infrared observations of the protostars IRAS 2A (JWST) and W33A (ISO), validating the predictive power of the model. Furthermore, the computational methodology employed predicts several chemically viable species below the current detection thresholds, highlighting targets for future observations. These results reinforce the role of ionizing radiation in the formation of ice chemistry in protoplanetary disks and demonstrate the usefulness of PROCODA in connecting laboratory astrochemistry with astronomical spectroscopy, particularly for the interpretation of molecular signatures in cold and sheltered environments.

## Abstract 2

We present a computational study of radiolytic chemistry and desorption dynamics in astrophysical ice composed of H_2_O : HCOOH (1 : 1), irradiated with Ni^11+^ ions at 46 MeV and 15 K.

## Graphical abstract

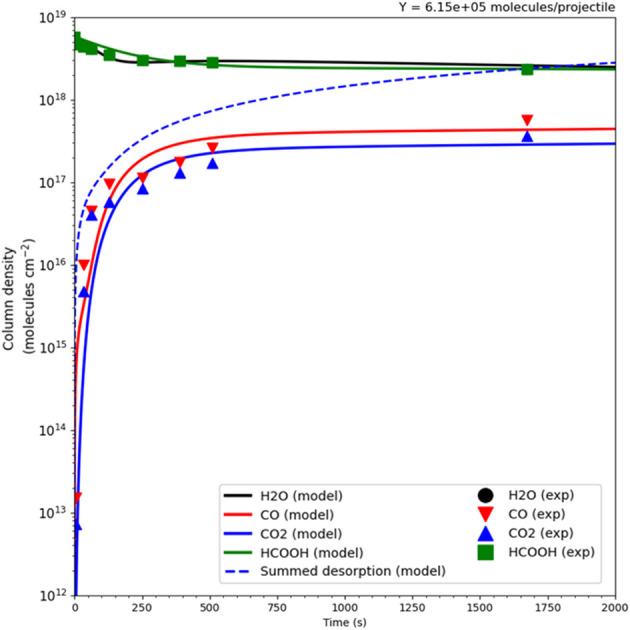

## 1 Introduction

The evolution of the universe is closely linked to the physicochemical processes occurring in diverse astrophysical environments, ranging from dense molecular clouds to protoplanetary disks. Simple molecules like hydroxyl (OH) and more complex species such as formic acid (HCOOH) and ethylene glycol (C_2_H_4_(OH)_2_) have been detected in various astrophysical environments^1^ indicating active chemistry even in regions of low temperature and density. Water (H_2_O), although structurally simple, plays a central role due to its polarity, phase versatility, and widespread presence in star-forming regions and interplanetary and interstellar medium. Its detection in solid, liquid, and gaseous phases is closely tied to the definition of the habitable zone in stellar systems and to the potential emergence of prebiotic environments.^2^ Understanding the mechanisms that govern the formation, evolution, and survival of these molecules requires a combined approach involving astronomical observations, laboratory experiments, and physicochemical modeling. The present study contributes to this field by investigating radiation-induced molecular processes in astrophysical ices, with a focus on analyzing the formation and destruction of species in simulated environments rich in HCOOH and H_2_O ices.

Formic acid is a key molecule in understanding the chemical pathways that lead to the formation of more complex organic species, such as esters and carboxylic acids, of possible prebiotic relevance. Its presence in astrophysical ices is significantly greater than in the gas phase approximately 50% more abundant, according to Andrade *et al.* (2008).^3^ This compound is formed predominantly by radiolysis and photolysis processes of precursors in ice sheets, which requires the development of theoretical models that integrate experimental data and chemical kinetics in a way that is coherent with astronomical observations. Computational modeling plays a central role in investigating the mechanisms of HCOOH formation and destruction under the action of ionizing radiation in astrophysical ices. The current numerical simulation allow the estimation of effective reaction coefficients, the temporal evolution of molecular abundances, and chemical equilibrium conditions, facilitating the interpretation of laboratory and observational data. Throughout this work, references to “irradiation” or “radiation processing” specifically refer to high-energy heavy ion interactions, unless otherwise indicated. The present modeling framework is tailored to such ionizing agents, consistent with experimental calibration conditions. It is worth noting that, in the present irradiated ice system, the so-called “chemical equilibrium phase” is more accurately described as a radiation-induced dynamic steady state, in which molecular formation and destruction rates become approximately balanced.

The investigation of the processes that lead to the formation and destruction of molecules in astrophysical environments requires, in addition to observations and laboratory experiments, the use of computational models that rigorously simulate the chemical reactions induced by radiation under conditions analogous to those of interstellar space. This approach is especially relevant for molecular ices that cover dust grains in cold and dense regions of the interstellar medium, where accumulation, irradiation and sublimation processes occur, resulting in significant chemical complexification.^4,5^ Among the complex molecules of astrophysical interest, formic acid (HCOOH) stands out for its recurrent detection in several cosmic environments, including dense molecular clouds, protoplanetary disks and envelopes of star-forming regions.^6,7^

Laboratory experiments have shown that UV irradiation of H_2_O : CO ices lead to the efficient formation of HCOOH at low temperatures (20–40 K), with reactions involving intermediates such as HCO and OH.^8^ Similarly, irradiation with 5 keV H^+^ ions also induce the formation of HCOOH and CO_2_ in CO : H_2_O ices at 10 K, with HCOOH remaining stable up to 150 K *e.g.* Bennett *et al.*^9^ (2011). Studies with more energetic ionizing radiation (46 MeV ^58^Ni^11+^) indicate that the presence of H_2_O in the ice significantly increases the rate of HCOOH destruction and favors the production of CO and CO_2_ as main fragments *e.g.* Bergantini *et al.*^10^ From an observational point of view, HCOOH has been detected in the gas phase in several hot cores, such as Orion KL and Sgr B2(N), with abundances consistent with thermal release of ice and not with *in situ* gas formation.^11^ Furthermore, it was identified in the coma of comet C/2014 Q2 (Lovejoy), reinforcing its solid origin and subsequent sublimation.^12^

In this research, we present a computational study to understand the chemical evolution of H_2_O : HCOOH (1 : 1) ice at 15 K under high-energy heavy ion irradiation, using the PROCODA code. Laboratory infrared data were obtained from Bergantini *et al.*^10^ In recent years, the PROCODA code has been widely used to investigate the chemical evolution of irradiated astrophysical ices under laboratory conditions. This work is an extension of previous work on the investigation of chemical evolution of pure HCOOH ice irradiated by swift ions.^13^ Here, we employ a chemical network containing 1631 reactions involving 73 molecular species to map time-dependent abundances, effective rate coefficients (ERCs), desorption rates, and equilibrium chemistry. Here, we consider 4 molecules detected in the experiments, also named as observed species (HCOOH, H_2_O, CO_2_, and CO) and 69 other molecules expected to participate in the reactions but not detected in the experiments for the chemical reaction network.

It is worth noting that while astrophysical ices are typically richer in H_2_O than in HCOOH, the H_2_O = 1 : 1 ice employed here was chosen to maximize the detectability of radiolytic products and to enable a detailed investigation of carbon chemistry induced by energetic processing (*e.g.* Gibb *et al.*^46^ 2000). Such a composition retains the essential matrix effects of water, including hydrogen bonding, energy dissipation, and radical-mediated chemistry, while providing sufficient precursor abundance for the identification of complex daughter species of astrochemical and astrobiological interest.

The current employed computational tool has provided valuable insights into the formation of unobserved (yet theoretically expected) species, the underlying reaction kinetics responsible for their production, the derivation of effective rate coefficients (ERCs) and desorption parameters, as demonstrated by Pilling *et al.*^14–17^ and Carvalho *et al.*^18,19^ More recently, PROCODA has also been employed to evaluate the temperature dependence of irradiation-induced chemistry in pure H_2_O ice^20^ and also at CO^21^ and HCOOH ices.^13^ Furthermore, the code was applied to simulate the chemical evolution of several binary ice mixtures under energetic processing, including H_2_O : O_2_, H_2_O : N_2_, H_2_O : CO_2_ and CO : CO_2_ mixed ices.^22–25^ In laboratory experiments and astronomical observations, some molecules cannot be detected for three main reasons: (1) low signal (below the detection limit); (2) the species, although occasionally present, does not have a permanent electric dipole moment and is therefore undetectable in the IR; (3) the lines/bands of this species in the IR are convoluted/mixed with other lines/bands from other species.

Section 2 describes the employed experimental dataset and the computational methodology. The main results including details on the best-fitting models are presented in Section 3 with highlights in molecular abundances at chemical equilibrium, and in desorption yields. In Section 4, we discuss the astrophysical implications highlighting the relevance of the use of the calculated ERCs in the astrochemical models and the relevance of this study for the understanding of ice chemistry of towards the protostars W33A and IRAS 2A. Finally, Section 5 contains the main conclusions and closing remarks.

## 2 Methodology

In this section, we describe the experimental data employed in the present study, including the procedures for data acquisition and processing, as well as the computational modeling strategy. The experimental dataset corresponds to mid-infrared spectra of H_2_O : HCOOH (1 : 1) ice mixtures irradiated under astrophysically relevant conditions, originally reported by.^10^ To simulate the chemical evolution of the irradiated ices, we employed the PROCODA code^13–17^ which numerically determines effective reaction rate coefficients, chemical abundances and desorption parameters by fitting the model output to experimental abundances.

### 2.1 The experimental data

The employed experimental data was obtained from Bergantini *et al.*^10^ (2014), by Fourier transform infrared (FTIR) absorption spectroscopy analysis of a H_2_O : HCOOH ice, with a ratio of 1 : 1, processed by 46 MeV ^58^Ni^11+^ ions at 15 K at Ganil Laboratory, France. Pure water and formic acid gases were mixed and deposited over potassium bromide (KBr) substrate inside an ultra-high vacuum (UHV). The ice thickness was calculated by the authors and considered to be 7.4 µm. Infrared spectral analysis revealed that the primary molecular products formed upon irradiation were CO and CO_2_, along with other, more complex species that were not quantified. The authors monitored the chemical evolution of only four species during the irradiation of the ice: H_2_O, HCOOH, CO, and CO_2_. Based on the experimental data, the destruction cross section of formic acid was estimated to be 2.4 × 10^−13^ cm^2^. Using this value, along with the estimated flux of galactic cosmic rays in dense interstellar environments, the half-life of HCOOH in the interstellar medium was calculated to be approximately 8 × 10^7^ years. The experimental results and parameters are summarized in Table 1.

**Table 1:** Experimental parameters used as input to the PROCODA code for HCOOH : H_2_O (Bergantini *et al.*, 2014)^10^

Parameters	Value
Employed ion beam	46 MeV ^58^Ni^11+^
Radiation flux (*Φ*)	2.0 × 10^9^ ions cm^−2^ s^−1^
Average projectile energy	46 × 10^6^ eV
Initial column density of ice	5.8 × 10^18^ molecules cm^−2^
Sample thickness	7.4 × 10^−4^ cm
Sample density	1.11 g cm^−3^
Sample area	∼0.6 cm^2^
Total desorption yield	7.0 × 10^4^ molecules per ion
Sample temperature	15 K

The construction and theoretical validation of the chemical network were further supported by complementary irradiation studies relevant to energetic particle and heavy cosmic-ray processing of astrophysical ices. In particular, the experiments under UV and proton irradiation, have provided important constraints on the formation pathways and stability of formic acid in CO : H_2_O and CO_2_ ices that are also applicable to ion-driven chemistry.^3,8,9^ Additionally, laboratory and space observations^12,26,27^ were used to identify chemically plausible product species formed during heavy-ion processing of mixed ices. This comparative, multi-source approach strengthens the robustness of the chemical network and enhances its applicability to astrophysical environments dominated by cosmic-ray-induced ice processing.

### 2.2 The PROCODA code

The main analysis of experimental data was performed employing the PROCODA code (Program for solving Coupled Differential equations in Astrochemistry). Briefly, the current model, 73 chemical species: 4 experimentally observed species (HCOOH, H_2_O, CO_2_ and CO) and 69 predicted (non-observed) species (H, H_2_, C, CH, CH_2_, CH_3_, O, CH_4_, OH, H_2_O, C_2_, CHCH, C_2_H_3_, CO, CH_2_CH_2_, HCO, C_2_H_5_, H_2_CO, CH_3_CH_3_, CH_2_OH, CH_3_O, O_2_, CH_3_OH, HO_2_, H_2_O_2_, C_3_, C_3_H_3_, C_2_O, C_3_H_4_, C_2_OH, C_3_H_5_, H_2_CCO, HCCOH, CO_2_, CH_2_CHOH, CH_2_OCH_2_, CH_3_CHO, CH_3_CH_2_CH_3_, HOCO, HOCHCH_3_, CH_2_OO, HCOOH, H_2_CO_2_, CH_3_CH_2_OH, CH_3_OCH_3_, O_3_, CH_2_OHOH, CH_3_OOH, C_3_O, C_3_OH, C_2_O_2_, CH_3_COCH_2_, CH_3_COCH_3_, C_2_H_3_O_2_, CO_3_, CH_3_COOH, HCOOCH_3_, OHCH_2_CHO, OHCHCHOH, CH_3_CHOHCH_3_, CH_3_OCH_2_CH_3_, CH_3_CH_2_CH_2_OH, HCO_3_, CH_3_OCHOH, H_2_CO_3_, CH_3_CHOHOH, CH_3_OCH_2_OH, CH_3_OOCH_3_, HOCH_2_CH_2_OH, C_3_O_2_, C_2_O_3_, OHCCOOH e CH_3_CH_2_CHOHOH) which are linked by 1631 coupled reactions (see SI online at https://doi.org/10.5281/zenodo.18379529). It is important to note that the absence of a given species in the IR spectra does not necessarily imply its absence in the irradiated ice. Some molecules, such as homonuclear species (*e.g.*, H_2_, and O_2_), are infrared inactive and therefore cannot be detected by infrared absorption spectroscopy. In addition, some species may exhibit absorption bands that are strongly overlapped by more intense features from other molecules, while others may be present at abundances below the instrumental detection limit or possess intrinsically weak infrared band strengths. Consequently, some of the species predicted by the chemical model may remain experimentally undetected despite participating in the underlying ice chemistry. Future measurements including QMS measurements during ice radiolysis my help to add constrain in these desorption modeled values.

The evolution of each species is modeled using differential equations that describe changes in column density due to dissociation, reactions, and radiation-induced desorption, with reaction rates expressed as Effective Rate Coefficients (ERCs), adapted from gas-phase to solid-phase kinetics. It is important to emphasize that PROCODA uses a chemically plausible reaction network previously validated in other contexts, allowing projections in chemical equilibrium based on coupled reaction kinetic laws and formation/destruction rules. The four-species calibration (the observed species in the experiments) serves to adjust the effective rate coefficients (ERCs), but the complete network is constructed from bimolecular, and radiation-induced reactions, based on thermodynamic data and specialized literature. This approach allows for a comprehensive analysis of chemical kinetics under the specified experimental conditions related to space conditions. See details at Pilling *et al.*^14–17^ See also PROCODA online database at https://www.univap.br/procoda. It is important to note that the chemical network extends beyond the four species directly monitored experimentally, allowing the model to infer the abundance evolution of chemically connected but unobserved species (predicted species). Consequently, the predicted abundances of unobserved species should be interpreted as constrained model estimates rather than direct experimental measurements.

The PROCODA computational model is based on six core assumptions to ensure physicochemical consistency in simulations of irradiated astrophysical ices: (1) a fixed set of chemical species including species observed in the experiments and expected species witch the formation is kinetically and thermodynamically possible in the simulated environment, even if they have not yet been experimentally detected due to spectroscopic limitations as mentioned in the introduction the reasons. The species set corresponds to a subset of molecules with greater astrophysical relevance or observational support, prioritizing physicochemical viability. Moreover, the 69 predicted (expected) species were considered from several laboratory and observational studies that have investigated the chemistry of formic acid containing ices (or related ices)^3,9,10,12,13,28^ and also consulting possible reactions from UMIST and KIDA databases.^29,30^

(2) The employed reactions network was set by employing a Python-based module developed by our group (RGEN-PROCODA), which systematically enumerates all possible radiolytic dissociation channels and bimolecular reactions involving the 73 species considered. Additionally, this module applies two physicochemically motivated filters: (i) an endothermicity criterion, which excludes energetically unfavorable pathways, and (ii) a diffusion-based criterion, which eliminates reactions whose reactants exhibit insufficient average mobility (*e.g.*, large species) within the ice matrix. Only the most thermodynamically and kinetically plausible reactions pass these filters and are retained in the final reaction network used in the calculations. The reaction network comprises also a limited number of experimentally supported reactions^31^ avoiding speculative mechanisms. It is worth noting that only neutral and radical chemistry is included for computational issues.

(3) The ERCs are thermochemically ranked, favoring exothermic reactions likely to occur at low temperatures (this occurs only at phase 1 calculation as we discuss further). (4) Atomic mass is strictly conserved across all reactions, including desorption processes. (5) Desorption parameters are adjusted initially in attempt to reproduce experimental values. (6) At high fluences, the system is assumed to reach chemical equilibrium, consistent with experimental evidence and ensuring realistic concentration limits (see also discussion at Pilling *et al.*^14,15^ and Hudson & Moore.^32^

The employed equation to describe the chemical evolution of a given species in the code (the coupled chemical network system) is given by:1
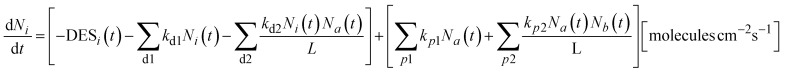
where *N*_*i*_ as the column density of a given species *i*, in units of molecules cm^−2^, d*N*_*i*_*/*d*t* is the variation in column density over time *t*, the *k* values are the ERCs for the different reactions and *L* indicates the average sample thickness in units of cm. The term DES_*i*_(*t*) = *k*_des,*i*_ Ω_*i*_ (*t*) *N*_*i*_(*t*) is the differential estimate of the number of atomic species that are desorbed from the ice into the gas phase per cm^2^ and per second due to incident radiation, which also depends on the intrinsic desorption rate (*k*_(des,*i*)_), in units of s^−1^, and the dimensionless surface coverage of species *i* as a function of time (*Ω*_*i*(*t*)_). The equation also has the following parameters: *k*_d*1*_ and *k*_d*2*_ indicate the processes of destruction (consumption), representing the direct dissociation reactions induced by radiation and have units of s^−1^, and the values *k*_*p*1_ and *k*_*p*2_ indicate the processes of production of a given species *i,* representing the bimolecular collision reactions induced by radiation, in units of cm^−2^ molecules per s. The *N*_*a*_ e *N*_*b*_ values indicate the column densities of species *a* and *b*, respectively, which take part in the reaction to produce or consume the respective species *i*, in units of molecules cm^−2^.

It should be noted that the experimental desorption yield represents an estimate derived only from species that can be identified and quantified by IR spectroscopy. In contrast, the chemical network employed by PROCODA includes both experimentally observed species and additional species predicted by the model, some of which may be infrared inactive, spectrally blended, or present below the detection limit. Consequently, differences between the modeled and experimental desorption yields are expected and partly reflect the incomplete experimental inventory of species present in the irradiated ice. Moreover, the direct comparison between experimentally estimated desorption and modeled desorption should be interpreted with caution, since it may reflects both model uncertainty and the inherent limitations of the experimental detection method.

The complete network used in this study is documented in SI online, including detailed information for each reaction: type (direct or bimolecular dissociation reactions), reaction class (dissociation, recombination, abstraction, *etc.*), estimated effective rate coefficient (ERC). All reactions were checked for stoichiometric consistency and thermodynamic plausibility. Reactions violating atomic conservation or involving improbable intermediates were excluded. The computational strategy employed numerical integration using adaptive step solvers with stability control, and convergence was evaluated based on equilibrium detection criteria (slope similarity and mass conservation). This methodology ensures that the predicted abundances represent physically feasible stationary states under continuous irradiation. By combining experimental calibration, network validation, and rigorous numerical integration, the PROCODA framework provides a reproducible and physically consistent platform for modeling astrochemical ice chemistry under high-energy processing.

As discussed in Pilling *et al.*,^17^ the modeling proceeds in two phases. In Phase 1 (“thermochemistry-driven phase”), the ERCs are initially ranked according to thermochemical data in the gas phase, ensuring that more exothermic reactions proceed faster than less exothermic (or endothermic ones) reactions within the same group of reactants. In Phase 2 (“weakly relaxed system phase”), we remove the thermochemistry-based constraints on ERCs and refine the model's parameters around the best solution from Phase 1, allowing a closer fit to the experimental data. This two-step approach reduces the degeneracy of possible solutions and ensures consistency with the real chemical pathways described in our reaction set. In short, using thermochemical data to guide ERCs in Phase 1 produces a more realistic chemical model, preventing largely endothermic reactions from being faster than exothermic ones involving the same reactants. This methodology allows PROCODA to model several competitive and simultaneous complex processes like desorption, molecular formation, and destruction more realistically, making it a valuable tool for astrochemical studies.

In the search for the best solution for coupled chemical systems, the score function (SF) was used in the minimization algorithm following eqn (2).2
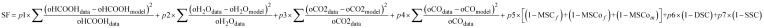


The dimensionless parameters, *p*1 to *p*7, serve as weights for each term to find the best solution during computational minimization processes. The equation compares the observed column density values of certain species, obtained from the interpolated experimental data, with their respective values calculated by the model. The chi-square function *x*^*2*^ is a parameter obtained by comparing the experimental column density of observed molecules (HCOOH, H_2_O, CO, CO_2_) with the modeled column density. It is important to note that the *x*^2^ value can be obtained by setting *p*1 = *p*2 = *p*3 = *p*4 = 1 and *p*5 = *p*6 = *p*7 = 0.

The MSC_f_ parameter (model mass similarity criterion) compares the initial and final column mass of the modeled system, ensuring mass conservation throughout the simulation. It is based on the principle that the sum of atomic masses in reactants must equal that in products—an essential foundation of the PROCODA scoring function. MSCo_f_ and MSCo_m_ represent the similarity between the experimental and modeled column masses at the final and midpoints of the simulation, respectively. Together, these yield the mass similarity criterion MSC = (MSC_*f*_ + MSCo_*f*_ + MSCo_*m*_) × 100%/3, which assesses the model's consistency with observed data. The DSC (desorption similarity criterion) compares the experimental and modeled total desorption yields. The SSC (slope similarity criterion) evaluates whether the model reaches chemical equilibrium, defined by stable concentrations and balanced reaction rates at high radiation fluence, as described in Pilling *et al.*^14–17^

The code, although it has some limitations in the equations used and species considered, simulates a vast number of reactions occurring simultaneously in the system and calculates the rate coefficients of these reactions. The code calculates the abundances of species not observed in the experiment but predicted. The code provides valuable values on the radiation-induced desorption rates and information on the branching ratios of reactions in groups that belong to a class of reactions involving a common reagents (see SI online). To guarantee the best-fit solution among the large number of possible solutions and to reduce model degeneracy, several physical and chemical constraints are imposed. These include: (i) conservation of mass throughout the simulation, (ii) convergence toward a chemical equilibrium phase (more precisely, a radiation-induced dynamic steady state) at large exposure times, and (iii) agreement between the calculated total desorption and the experimentally estimated value. A detailed description of the procedures adopted to minimize model degeneracy and an assessment of the associated methodological uncertainties (estimated to be on the order of 20%) are provided in Pilling *et al.*^14–17^

It is important to clarify that the ERCs and desorption parameters derived in the best-fit model are also in agreement with the high linear energy transfer (LET) conditions characteristic of the employed fast heavy ion irradiation (see also the experimental data Bergantini *et al.*^10^). Consequently, the term “irradiation-induced chemistry” used throughout this manuscript should be interpreted as ion-induced radiolysis and does not encompass (specifically in this work) processes driven primarily by low-LET agents, such as UV photons, soft X-rays, or low-energy electrons, unless explicitly stated otherwise, although PROCODA in other works separately analyzes chemistry driven by other ionizing agents see Pilling *et al.*^14–17^ Extrapolation of simulation results to the broader cosmic ray spectrum should be done with caution. Galactic cosmic rays encompass a wide range of ion types (from protons to iron nuclei) and energies (from keV to GeV per nucleon). In this context, the current results are more representative of intermediate cosmic ray analogs, MeV per nucleon, which dominate energy deposition in dense astrophysical environments.

Although PROCODA has been successfully employed in studies involving different types of ionizing agents such ions, electrons and ionizing photons, and varying fluence levels (*e.g.* ref. 13–24), the code does not incorporate photo processing modules and interaction cross-sections modules. The integration of photon-driven reaction networks, including photoabsorption cross-sections and photodesorption yields, is currently under development and will be addressed in future versions of the model. Until then, its use should remain limited to ion-driven processing scenarios, and all conclusions presented here are strictly valid for heavy ion-induced ice chemistry at cryogenic temperatures. This clarification is particularly relevant when comparing our results with studies involving soft X-ray photodesorption, such as those reported by Basalgète *et al.*^28^ It should be noted that, although both X-rays and energetic ions can induce molecular dissociation and chemical evolution in astrophysical ices, heavy ions deposit energy through dense ionization tracks and secondary-electron cascades, leading to significantly more efficient sputtering and large-scale ice destruction than the localized electronic excitations typically produced by X-ray irradiation.

To enhance the transparency and reproducibility of our approach, it is important to highlight that the PROCODA code solves coupled differential equations based on a reaction network previously validated in different astrochemical scenarios. The reaction rates, expressed as ERCs, are thermodynamically and kinetically weighted, with exothermic and barrierless reactions favored at 15 K. The use of a score function incorporating chemical equilibrium, desorption match, and mass conservation ensures convergence toward a solution that reflects both the experimental dataset and the expected physicochemical behavior of irradiated ices. Additionally, each reaction pathway is dynamically evaluated based on the temporal availability of reactants and the evolution of radicals and intermediates, allowing the simulation to reflect the progressive nature of radiation-induced chemistry. While the code does not solve the full microphysics of energy dissipation (*e.g.*, track structure, electronic excitation), it effectively bridges between laboratory-scale processes and the integrated molecular outcomes observed in astronomical environments. Finally, it is worth noting that the present chemical network is restricted to neutral molecules and radicals and does not explicitly include ionic or electronically excited species. Although such species are expected to be produced during the initial stages of heavy-ion radiolysis, their reaction pathways and kinetic parameters in condensed astrophysical ices remain poorly constrained experimentally. Furthermore, rapid charge neutralization, electron recombination, and de-excitation processes are expected to convert a significant fraction of these species into neutral radicals and molecules, whose chemistry is the primary focus of the current model.

## 3 Results and discussion

This section presents the main results obtained, highlighting the molecular abundances at chemical equilibrium. Subsection 3.1 discusses the best-fitting model, including the temporal evolution of the abundances of the 73 simulated species under irradiation. Subsection 3.2 analyzes the most relevant reactions in the formation and destruction of H_2_O, CO_2_, CO, H_2_CO, HCOOH, and CH_2_OH in irradiated molecular ices, based on the effective rate coefficients (ERCs) calculated by the PROCODA code. These species, detected in star-forming regions and protoplanetary disks by missions such as ISO and JWST, are particularly relevant from an astrochemical perspective. Subsection 3.3 addresses radiation-induced desorption in H_2_O : HCOOH (1 : 1) ices at 15 K, while subsection 3.4 examines the relative abundances reached at chemical equilibrium.

### 3.1 The best-fit model

**Fig. 1:**
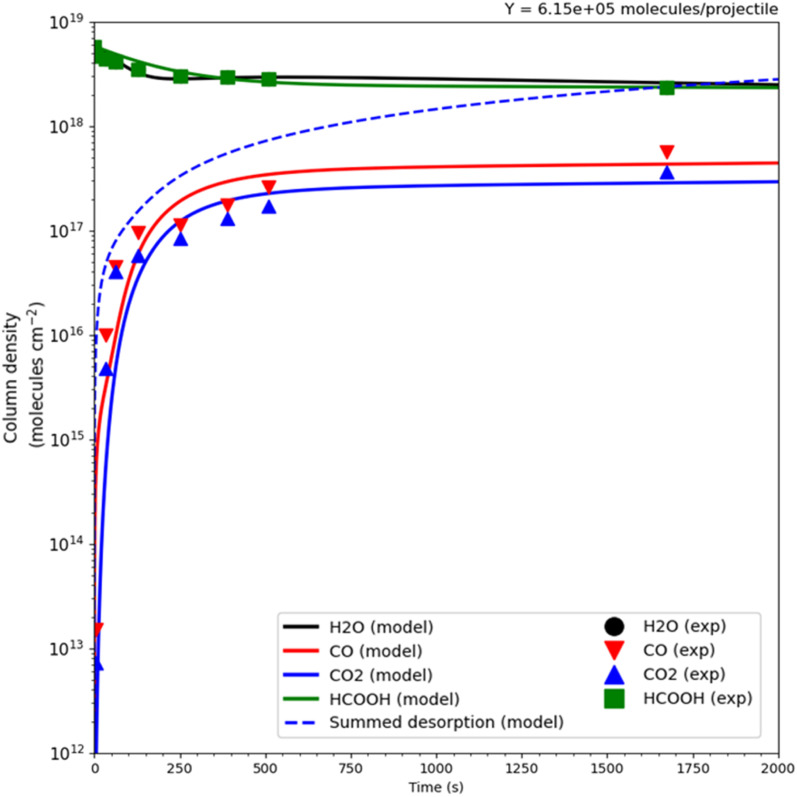
Time evolution of column densities of observed molecules obtained by best-fit models of H_2_O : HCOOH (1 : 1) ice irradiated at 15 K, using the PROCODA code. Experimental data points from Bergantini *et al.*^10^ are shown for selected molecules (H_2_O, CO, CO_2_, and HCOOH). The simulation reveals the chemical equilibrium phase being reached around 2000 seconds.

Fig. 1 presents the modeled column densities (in molecules cm^2^) for the observed species in a H_2_O : HCOOH (1 : 1) ice mixture irradiated at 15 K by 46 MeV ^58^Ni^11+^ ions. The experimental data of Bergantini *et al.*^14^ are also indicated in the figure by discrete symbols. The chemical equilibrium phase, which, according to the PROCODA simulation, is reached in approximately 2000 s (horizontal plateau at large exposure time). Although the full simulation spans 5000 s, the selected snapshot emphasizes the equilibrium condition, where the rate of formation and destruction of chemical species becomes stationary. The model shows very good agreement with the experimental data. The total desorption yield (*Y*) predicted by the model was 6.1 × 10^5^ molecules per projectile, approximately one order of magnitude higher than the value estimated by the authors in the experimental measurements. This enhancement may be attributed to the methodology's ability to monitor species that were not mapped during the experiments.

**Fig. 2:**
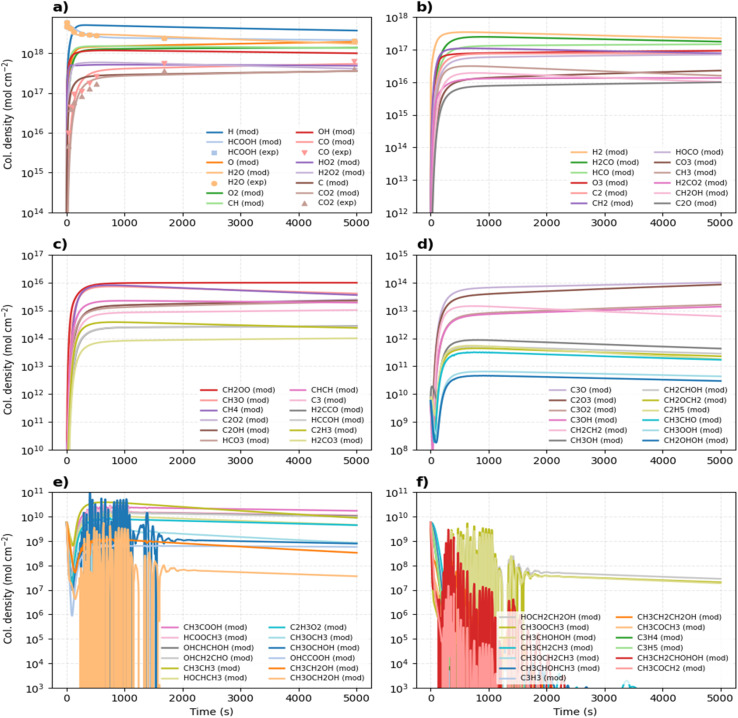
Time evolution of column densities for 73 chemical species resulting from irradiation of H_2_O : HCOOH (1 : 1) ice, as calculated by the best-fit simulation using the PROCODA code. Species are grouped in panels (a) to (f) in order of decreasing abundance at chemical equilibrium, highlighting their relative chemical importance. Panels (e) and (f) clearly indicate non relevant species. Simulations highlight the formation and stabilization of radicals, stable products, and complex organics in irradiated astrophysical ices.

The best-fit modeled column densities for the 73 employed species are presented in Fig. 2 (Panels a–f), where symbols once more indicate experimental data. The figure illustrates the formation and accumulation of several key molecular products. Species such as CO, CO_2_, H_2_O, CH_3_OH, H_2_CO, HCO, CH_2_OH, and OH appear as dominant components, reflecting their high chemical stability and significant reactivity under ionizing radiation in cryogenic environments. Early-time fluctuations, particularly among reactive intermediates like radicals (*e.g.*, CH_3_, CH_2_, OH), indicate rapid chemical dynamics, as these species are swiftly converted into more stable products through reaction chains. The arrangement of species in the figure follows their relative importance in the modeled system. Panels (a) through (d) display species deemed chemically relevant, organized from highest to lowest abundance, while panels (e) and (f) group those with negligible contributions exhibiting fluctuations in column densities, reflecting logarithmic values close to 0, and not presented in a linear fashion. The *y*-axis was adjusted accordingly to reflect this relevance hierarchy, emphasizing the abundance gradient from dominant to marginal species. It is important to note that the high modeled abundances of species such as H_2_O, CH_4_, and H_2_CO result from the efficiency of radiation-induced chemistry under simulated laboratory conditions. These results should not be interpreted as indicating that radiolysis is the dominant formation pathway for these molecules in the interstellar medium, where alternative processes, such as grain-surface hydrogenation, are known to play a critical role *e.g.*, Dulieu *et al.*^33^ and Potapov *et al.*^34^

A comprehensive comparison with previous experimental and theoretical studies in the field of ice astrochemistry underscores the broader context and supports the interpretations presented herein. Extensive laboratory investigations, particularly on water-rich ices subjected to energetic processing, have been reported by multiple research groups worldwide. The Cosmic Ice Laboratory at NASA's Goddard Space Flight Center has documented systematic radiolysis studies of H_2_O and other astrophysical ice analogues under proton and electron irradiation, demonstrating the formation of complex organic species and identifying reaction pathways relevant to both interstellar ices and cometary environments.^35–37^

Complementary experiments by Moore, Hudson, and collaborators^35,36^ have elucidated the formation of key ions such as OCN^−^ and the effect of matrix composition on product yields, providing essential benchmarks for model calibration. Specifically for formic acid, the work of Bergantini *et al.*^10^ remains among the few quantitative experimental studies involving heavy ion bombardment of H_2_O : HCOOH ice analogs, revealing dissociation cross sections, half-life estimates under cosmic ray analogs, and resulting product distributions dominated by CO and CO_2_ production.

Notably, the modeled trends for key species, such as CO, CO_2_, H_2_O and HCOOH, are consistent with experimental data, reinforcing the model's predictive accuracy. These results underscore the efficiency of PROCODA in reproducing radiation-induced molecular synthesis under astrophysical ice conditions. The ability to simulate both transient and long-lived species within a unified kinetic framework is crucial for understanding the chemical evolution of ices in environments such as dense molecular clouds, protoplanetary disks, and icy solar system bodies.

The correspondence between model and experiment is particularly good for the most abundant species, validating the kinetic network implemented in PROCODA. The lightest species, such as H_2_, CO, CO_2_, OH, HCO and H_2_CO, exhibit the highest column densities, due to their rapid formation from primary dissociation pathways of HCOOH and water. These molecules have low molecular weight, simple structure and high mobility in ice, which favors both their initial formation and their stabilization over time. Furthermore, as they are direct products of molecular fragmentation processes or intermediates in highly exoenergetic reactions, these species are generated in large quantities in the early stages of the simulation and maintain high concentrations throughout the irradiation time. In parallel, the model also simulates the reformation of initially present species such as H_2_O and HCOOH, through reaction chains involving radicals and molecular fragments generated by radiolysis (*e.g.*, OH + H → H_2_O or CO + OH → HCOOH). This reflects the dynamic balance between molecular destruction and reassembly, a behavior consistently observed in laboratory experiments with ion-irradiated ices. Therefore, the apparent increase in these parental species over time should be interpreted as a result of chemical recycling rather than primary synthesis. Their predominance is consistent with experimental observations of irradiated ices, reinforcing their central role in radiation-induced chemistry in cold astrophysical environments.

An important outcome of this study is the distinction between relevant and non-relevant species among the initial chemical components used to model the experimental data of irradiated H_2_O : HCOOH (1 : 1) ice. The relevant species (the species with larger contribution in the chemical dataset) for this ices were: H, H_2_, C, CH, CH_2_, CH_3_, O, CH_4_, OH, H_2_O, C_2_, CHCH, C_2_H_3_, CO, CH_2_CH_2_, HCO, C_2_H_5_, H_2_CO, CH_3_CH_3_, CH_2_OH, CH_3_O, O_2_, CH_3_OH, HO_2_, H_2_O_2_, C_3_, C_2_O, C_2_OH, H_2_CCO, HCCOH, CO_2_, CH_2_CHOH, CH_2_OCH_2_, CH_3_CHO, HOCO, CH_2_OO, HCOOH, H_2_CO_2_, O_3_, CH_2_OHOH, CH_3_OOH, C_3_O, C_3_OH, C_2_O_2_, CO_3_, CH_3_COOH, HCOOCH_3_, OHCH_2_CHO, OHCHCHOH, HCO_3_, H_2_CO_3_, C_3_O_2_, C_2_O_3_ with represents 72% of the total employed species in this study. The numerical values for the evolution of column densities and desorption (normalized by parent species) obtained by the best-fit model can be found in the SI online material.

### 3.2. Dominant formation and consumption reaction pathways for selected species (H_2_O, CO_2_, CO, H_2_CO, HCOOH, and CH_3_OH)

Based on modeling performed with the PROCODA code, it was possible to identify the main production and consumption pathways among the 73 species formed in the H_2_O : HCOOH (1 : 1) ice. In particular, the reactions associated with the species H_2_O, CO_2_, CO, H_2_CO, HCOOH, and CH_2_OH stand out, widely detected in star-forming regions such as the protostars IRAS 2A (JWST) and W33A (ISO). These results show that the chemical network is dominated by radiation-induced dissociation processes and subsequent bimolecular reactions, involving both stable species and highly reactive radicals, such as HCO and CH_2_OH, which play a central role in the chemical evolution of the ice.

Table 2 presents the dominant production and consumption reactions for the species H_2_O, CO_2_, CO, H_2_CO, HCOOH, and CH_3_OH in irradiated HCOOH : H_2_O ice, as modeled by the PROCODA code. The analysis considers two irradiation regimes: low dose (beginning of the simulation) and high dose (at the end of the experiment with the ice highly irradiated when it reaches chemical equilibrium phase). The table presents also the respective effective rate coefficients (ERCs), which dominate the kinetics of these six species in the irradiation regime. Reaction numbers and full details can be found in SI online.

**Table 2:** Dominant two-most formation and consumption reaction pathways for selected species (H_2_O, CO_2_, CO, H_2_CO, HCOOH e CH_3_OH) computed with the PROCODA code for the studied irradiated H_2_O : HCOOH ice. Note: R in the reaction represents

	Low-dose irradiated ice (beginning of experiment) [reaction label; effective reaction rate^a^^,^^b^]	Highly irradiated ice (CE phase) [reaction label and effective reaction rate^a^^,^^b^]
**H**_**2**_ **O**		
Formation pathway	H + OH → H_2_O [r101; *k* = 3.61 × 10^−24^]	H + OH → H_2_O [r101; *k* = 3.61 × 10^−24^]
1st reaction		
2nd reaction	H_2_O_2_ + R → O + H_2_O [r112; *k* = 1.43 × 10^−02^]	H_2_O_2_ + R → O + H_2_O [r112; *k* = 1.43 × 10^−02^]
Consumption pathway	H_2_O + R → H + OH [r100; *k* = 2.95 × 10^−03^]	O + H_2_O → H_2_O_2_ [r113; 3.96 × 10^−24^]
1st reaction		
2nd reaction	H + H_2_O → H_2_ + OH [r118; *k* = 4.81 × 10^−25^]	H_2_O + R → H + OH [r100; 2,95 × 10^−03^]

**CO**_**2**_		
Formation pathway	O + CO → CO_2_ [r160; *k* = 2.35 × 10^−24^]	CO + O_2_ → O + CO_2_ [r205; *k* = 3.44 × 10^−24^]
1st reaction		
2nd reaction	C + O_2_ → CO_2_ [r146; *k* = 2.98 × 10^−24^]	O + H_2_CO → H_2_ + CO_2_ [r1023; *k* = 7.32 × 10^−24^]
Consumption pathway	CO_2_ + R → O + CO [r159; *k* = 2.21 × 10^−02^]	CO_2_ + R → O + CO [r159; *k* = 2.21 × 10^−02^]
1st reaction		
2nd reaction	CO_2_ + R → C + O_2_ [r145; *k* = 9.64 × 10^−03^]	H + CO_2_ → HOCO [r231; *k* = 2.77 × 10^−24^]

**CO**		
Formation pathway	C + O → CO [r142; *k* = 3.12 × 10^−24^]	CO_2_ + R → O + CO [r159; *k* = 2.21 × 10^−02^]
1st reaction		
2nd reaction	HCO + R → H + CO [r218; *k* = 3.59 × 10^−02^]	HCO + R → H + CO [r218; *k* = 3.59 × 10^−02^]
Consumption pathway	CO + R → C + O [r141; *k* = 8.46 × 10^−03^]	H + CO → HCO [r219; *k* = 3.81 × 10^−24^]
1st reaction		
2nd reaction	H + CO → HCO [r219; *k* = 3.81 × 10^−24^]	CO + R → C + O [r141; *k* = 8.46 × 10^−03^]

**H**_**2**_ **CO**		
Formation pathway	C + H_2_O_2_ → O + H_2_CO [r1068; *k* = 4.62 × 10^−24^]	H + HCO → H_2_CO [r221; *k* = 7.65 × 10^−24^]
1st reaction		
2nd reaction	H + HCO → H_2_CO [r221; *k* = 7.65 × 10^−24^]	C + H_2_O_2_ → O + H_2_CO [r1068; *k* = 4.62 × 10^−24^]
Consumption pathway	O + H_2_CO → H_2_ + CO_2_ [r1023; *k* = 7.32 × 10^−24^]	O + H_2_CO → H_2_ + CO_2_ [r1023; *k* = 7.32 × 10^−24^]
1st reaction		
2nd reaction	H + H_2_CO → CH_2_OH [r223; *k* = 1.22 × 10^−24^]	H + H_2_CO → CH_2_OH [r223; *k* = 1.22 × 10^−24^]

**HCOOH**		
Formation pathway	C + H_2_O_2_ → HCOOH [r324; *k* = 1.38 × 10^−23^]	C + H_2_O_2_ → HCOOH [r324; *k* = 1.38 × 10^−23^]
1st reaction		
2nd reaction	H + HOCO → HCOOH [r235; *k* = 2.88 × 10^−24^]	O + H_2_CO → HCOOH [r462; *k* = 2.31 × 10^−24^]
Consumption pathway	HCOOH + R → CH + HO_2_ [r355; *k* = 2.67 × 10^−03^]	HCOOH + R → CH + HO_2_ [r355; *k* = 2.67 × 10^−03^]
1st reaction		
2nd reaction	HCOOH + R → H_2_O + CO [r597; *k* = 1.77 × 10^−05^]	HCOOH + R → H_2_O + CO [r597; *k* = 1.77 × 10^−05^]

**CH**_**3**_ **OH**		
Formation pathway	CH_3_CHO + R → C + CH_3_OH [r318; *k* = 7.51 × 10^−02^]	CH_3_CHO + R → C + CH_3_OH [r318; *k* = 7.51 × 10^−02^]
1st reaction		
2nd reaction	CH_2_CHOH + R → C + CH_3_OH [r316; *k* = 1.10 × 10^−02^]	CH_2_CHOH + R → C + CH_3_OH [r316; *k* = 1.10 × 10^−02^]
Consumption pathway	CH_3_OH + R → CH_2_ + H_2_O [r371; *k* = 5.10 × 10^−03^]	O + CH_3_OH → CH_3_OOH [r472; *k* = 3.43 × 10^−24^]
1st reaction		
2nd reaction	CH_3_OH + R → CH_3_ + OH [r422; *k* = 4.29 × 10^−03^]	O + CH_3_OH → OH + CH_3_O [r1175; *k* = 2.14 × 10^−24^]

a Units for bimolecular reactions is molecules cm^−2^ s^−1^; units for dissociation reactions is s^−1^.

b The list of reactions is presented in the SI online.

For H_2_O production, the reaction H + OH → H_2_O (r101; *k* = 3.61 × 10^−24^) is the main pathway throughout the simulation range, favored by the high mobility of the reactants. The reaction H_2_O_2_ + R → O + H_2_O (r112; *k* = 1.43 × 10^−02^) also contributes significantly, especially in the irradiated steps. Regarding consumption, the reaction H_2_O + R → H + OH (r100; *k* = 2.95 × 10^−03^) is the main water dissociation pathway at the initial times. The reaction O + H_2_O → H_2_O_2_ (r113; *k* = 3.96 × 10^−24^) assumes dominance at the final time in chemical equilibrium due to the high availability of reactant O.

In the formation of CO_2_, the reaction O + CO → CO_2_ (r160; *k* = 2.35 × 10^−24^) begins to dominate due to its significant rate coefficients. The reaction CO + O_2_ → O + CO_2_ (r205; *k* = 3.44 × 10^−24^) begins to dominate at chemical equilibrium, with strong participation from the secondary reaction O + H_2_CO → H_2_ + CO_2_ (r1023; *k* = 7.32 × 10^−24^), both driven by the availability of oxygenated precursors. For CO_2_ consumption, the dominant reaction is CO_2_ + R → O + CO (r159; *k* = 2.21 × 10^−02^), while CO_2_ + R → C + O_2_ (r145; *k* = 9.64 × 10^−03^) maintains secondary relevance at early times.

The CO production is controlled by the reaction HCO + R → H + CO (r218; *k* = 3.59 × 10^−02^), with a significant contribution to production. However, in the final stages, in the chemical equilibrium phase, the reaction CO_2_ + R → O + CO (r159; *k* = 2.21 × 10^−02^) assumes dominance due to the high value of the rate coefficient. The reaction C + O → CO (r142; *k* = 3.12 × 10^−24^) is also relevant in the initial phases. In CO consumption, the main pathway is CO + R → C + O (r141; *k* = 8.46 × 10^−03^), followed by H + CO → HCO (r219; *k* = 3.81 × 10^−24^), in chemical equilibrium whose importance increases with the availability of atomic H.

For H_2_CO, formation occurs primarily *via* H + HCO → H_2_CO (r221; *k* = 7.65 × 10^−24^), a kinetically stable and efficient reaction pathway. The reaction C + H_2_O_2_ → O + H_2_CO (r1068; *k* = 4.62 × 10^−24^) contributes secondarily to the time to chemical equilibrium. For the consumption of H_2_CO, the most relevant reaction is O + H_2_CO → H_2_ + CO_2_ (r1023; *k* = 7.32 × 10^−24^), with a secondary contribution of H + H_2_CO → CH_2_OH (r223; *k* = 1.22 × 10^−24^) in the initial phases.

The formation of HCOOH is dominated by the reaction C + H_2_O_2_ → HCOOH (r324; *k* = 1.38 × 10^−23^), reflecting partial oxidation of carbon. The reaction O + H_2_CO → HCOOH (r462; *k* = 2.31 × 10^−24^) is also relevant, especially at high radiation doses. HCOOH is consumed primarily by HCOOH + R → CH + HO_2_ (r355; *k* = 2.67 × 10^−03^), and secondarily *via* HCOOH + R → H_2_O + CO (r597; *k* = 1.77 × 10^−05^).

For the formation of CH_3_OH, the reaction CH_3_CHO + R → C + CH_3_OH (r318; *k* = 7.51 × 10^−02^) stands out as the main formation pathway in all phases, with the reaction CH_2_CHOH + R → C + CH_3_OH (r316; *k* = 1.10 × 10^−02^) acting as a complementary route. The consumption of CH_3_OH is led by CH_3_OH + R → CH_2_ + H_2_O (r371; *k* = 5.10 × 10^−03^) at the initial times, while at the final times the reaction O + CH_3_OH → CH_3_OOH (r472; *k* = 3.43 × 10^−24^) assumes dominance with the contributions of the relatively significant rate coefficients and the availability of the reactant O contributing to the consumption.

### 3.3. Radiation-induced molecular desorption

The simulation of desorption processes in PROCODA is supported by empirical analogies and experimental calibration. Although the model is calibrated with Bergantini *et al.*,^10^ the desorption behavior of formic acid and related light species is also aligned with independent measurements. For example, Basalgète *et al.*^28^ reported significant photodesorption yields of HCOOH in X-ray irradiated ices, with yields on the order of 10^−3^–10^−2^ photon molecules. These results, while differing in the type of radiation (X-rays *vs.* heavy ions), corroborate the high sensitivity of formic acid to radiolytic processing. The inclusion of desorption as a kinetic term within the coupled equations allows the model to track temporal fluxes to the gas phase, integrating primary and secondary release channels. By considering measured and inferred desorption rates, the model bridges the gap between experimental observability and astrochemical relevance. The higher desorption yield predicted in our simulation compared to laboratory measurements may arise from cumulative contributions of light species (*e.g.*, H, OH, CO) that are difficult to detect experimentally but are chemically consistent with radiolysis results.

**Fig. 3:**
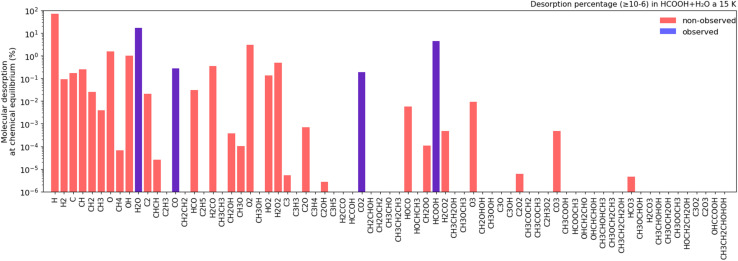
Radiation-induced molecular desorption at the chemical equilibrium phase, in percentage (only values higher 1 × 10^−6^%), obtained by the best-fit model. Blue and red bars represent species modeled by PROCODA, with blue indicating experimentally observed species and red indicating species predicted by the model.

Fig. 3 presents the radiation-induced molecular desorption at the chemical equilibrium phase, in percentage obtained by the best-fit model. This indicates the profile of molecular desorption to the gas-phase due to incoming radiation. The chemical equilibrium phase, obtained at large radiation fluences, correspond to the stage in which the rates of molecular formation and destruction are balanced, characterizing the chemical equilibrium phase. Only species with desorption yields greater than 10^−6^% were included, thereby emphasizing the most relevant contributors to the gas phase. The color coding distinguishes between experimentally observed species (blue bars) and those predicted exclusively by the model (red bars). This analysis provides key insights into which molecules are most efficiently released from the ice matrix under irradiation, contributing to the chemical enrichment of the surrounding gas in astrophysical environments. As shown in the figure, light and highly mobile species such as H, H_2_, OH, and O dominate the desorption process at chemical equilibrium. H_2_O also exhibits significant desorption, consistent with its high initial abundance and its structural role as the main component of the ice matrix. The detection of HCOOH, H_2_O_2_, and CO in the gas phase highlights the efficiency of radiation-induced fragmentation and subsequent recombination of molecular precursors under energetic processing. These outcomes are consistent with the reaction mechanisms incorporated in the best-fit PROCODA simulations. Overall, the results support the model's predictive capacity for identifying desorption pathways that align with both laboratory observations and astrophysical expectations.

Table 5 (in the appendix) presents the numerical values for the modeled desorption in the H_2_O : HCOOH (1 : 1) ice mixture at 15 K, as simulated using the PROCODA code. The values correspond to the chemical equilibrium stage, where formation and destruction rates are balanced. For each species, we report the equilibrium column density of desorbed molecules (molecules cm^−2^), the fractional desorption yields relative to the total abundance at equilibrium (%), and the average desorption rate (s^−1^). These values quantify the steady-state release of molecules into the gas phase under prolonged irradiation and provide constraints for interpreting observational data in cold, dense interstellar environments.

The methodology employed considers desorption processes as a type of chemical reaction within the chemical network, activated by the deposition of ionizing energy in the ice, promoting the release of fragments and molecules formed by radiolytic dissociation and subsequent recombination. The lightest and most volatile species (H, H_2_, CO, C, CH, OH, and H_2_O_2_) exhibit the highest rates of desorbed species, a result of their high mobility, molecular stability, and low binding energy with the ice matrix. These characteristics favor their efficient release even at low temperatures.

An important feature revealed in the modeling of the desorbed species is the dynamic behavior of radiation-induced desorption, where, at specific irradiation times, the desorption rates of certain species temporarily exceed those of others. This transient inversion reflects the evolution of the chemical network within the ice matrix, driven by radiolytic fragmentation, radical recombination, and the progressive depletion or accumulation of precursor species. During the temporal evolution of the H_2_O : HCOOH system under irradiation, a reversal in the predominance of the desorbed species was observed, with H_2_O dominating in the initial stages and the atomic species H becoming prevalent in the final stages, already at chemical equilibrium. This change can be explained by the thermodynamic and kinetic behavior of the species under energetic processing conditions, as modeled by the PROCODA code. This desorption simulation, based on fits of experimental data on the accumulated column density of the desorbed species, provides support for astrophysical interpretations of the volatile composition of the gaseous medium in cold regions, such as grain mantles in molecular clouds, the atmospheres of icy moons, and comets. Among the species formed and detected, formic acid, already identified in comets stands out.^38,39^

It is worth noting that several groups in Europe and North America have examined sputtering and radiolytic chemistry induced by ion impacts on water ice and mixed systems, highlighting the dependence of ion mass and energy on chemical yields. Some examples include the works of Bahr *et al.*^39,40^ These works investigated water ice radiolysis and sputtering of radiolytic products under ion irradiation relevant to outer solar system conditions demonstrating how ion energy and species affect sputtering yields and product entrapment in the ice matrix. Recently, some researchers have explored the influence of irradiation by energetic electrons and photon-induced chemistry on ice mixtures relevant to dense molecular cloud conditions, including electron-induced radiolysis of water and mixed ices and its impact on chemical formation.^41^

The code simulates the overall chemical dynamics of the system, calibrating reaction parameters to ensure that the total sum of desorbed species (including both observed and expected species) is compatible with the integrated experimental yields. This methodology provides greater robustness for modeling astrochemical environments, in which multiple reaction pathways and competing processes occur simultaneously. In the specific case of H_2_O : HCOOH mixed ice irradiated with the ^58^Ni^11+^ projectile, the PROCODA model estimates a total desorption yield of approximately 6.1 × 10^5^ molecules per ion, while the experimental value reported by Bergantini *et al.*^10^ is 7.0 × 10^4^ molecules per ion. This difference, approximately one order of magnitude, should not be interpreted as a modeling flaw, but rather as an inherent characteristic of PROCODA. The code was designed to reproduce the overall chemical dynamics of the system and the integrated desorption fluxes, without necessarily adjusting the behavior of individual species. Its strength lies in its ability to represent the total number of desorbed molecules, including light and volatile species such as H, OH, and H_2_, which may not be detected experimentally due to instrumental limitations. This systemic approach allows PROCODA to describe the collective desorption behavior more comprehensively than isolated experiments.

### 3.4. Molecular abundances in the CE phase

The chemical equilibrium (CE) phase corresponds to the stage in which the rates of formation and destruction of chemical species balance, leading to stable molecular abundances over time, despite continuous irradiation. This physicochemical condition is expected to occur in real astrophysical ices subjected to prolonged exposure to cosmic rays and UV photons. In astrochemical simulations using the PROCODA code Pilling *et al.*,^14–17^ the CE phase is clearly identified and plays a crucial role in defining the final molecular inventory of processed ices. Characterizing this phase allows the determination of effective rate coefficients, desorption yields, and the prediction of dominant species in cold interstellar environments, making it essential for the interpretation of laboratory and astronomical observations.

**Fig. 4:**
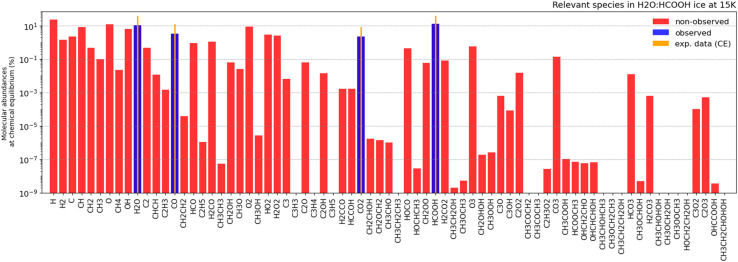
Molecular abundances at the chemical equilibrium phase, in percentage, for the relevant species (greater than 1 × 10^−9^%) obtained by the best-fit model. Blue and red bars represent species modeled by PROCODA, with blue indicating experimentally observed species and red indicating species predicted by the model. The thin orange bars represent original experimental data.

Fig. 4 presents the molecular abundances at chemical equilibrium (in percentage, logarithmic scale) of the 59 relevant species with a rate greater than 10^−9^ on the logarithmic scale in the best-fit model of H_2_O : HCOOH ice irradiated at 15 K. These values correspond to the final stage of the simulation using the PROCODA code, where the sum of the production and destruction rates of all species is equal. The blue bars indicate species that were experimentally detected in the irradiation experiments of Bergantini *et al.*,^10^ while the red bars represent species that were not detected in the laboratory but are predicted by the computational model as relevant products in the ice matrix. It is important to highlight that, over the blue bars, orange lines corresponding to the experimentally obtained molecular abundances are superimposed, allowing a direct comparison between the experimental results and those calculated by PROCODA in the chemical equilibrium regime. This overlap shows a certain agreement between the simulated data and the experimental data, reinforcing the robustness and accuracy of the model.

The inclusion of both observed and non-observed species enables a more comprehensive understanding of the radiation-induced chemical network, revealing hidden complexity in the ice's chemical evolution. The results also underscore the importance of including predicted but undetected intermediates and products when modeling astrochemical environments, especially under low-temperature and high-radiation conditions analogous to dense interstellar clouds or icy planetary surfaces. This detailed equilibrium profile provides insight into potential reservoirs of prebiotic molecules and supports the use of theoretical modeling as a complementary tool to experimental astrochemistry. It is worth notice that the relevant modeled species are essential for both the kinetic dynamics and the final molecular inventory, as they directly influence the chemical evolution of the irradiated system and the composition of the solid and gas phases in astrophysical environments. Their high abundance at equilibrium, combined with their reactivity and recurrent presence in several chemical pathways, makes them essential for modeling realistic astrochemical processes.

Table 6 (in the Appendix) presents the numerical values of molecular abundances at the chemical equilibrium phase for the H_2_O : HCOOH (1 : 1) ice mixture at 15 K, as obtained from the best-fit simulation using the PROCODA code. These values represent the steady-state concentrations after prolonged exposure to ionizing radiation, where the rates of molecular formation and destruction become balanced. The results reflect both experimentally observed molecules and predicted species, offering a comprehensive view of the chemical inventory within the processed ice. This quantitative analysis is essential for constraining astrochemical models and for interpreting infrared observations of irradiated ices in cold astrophysical environments.

### 3.5. Comparison with previous modeling of pure HCOOH ice

In this section, we present a comparative analysis between the current results and those reported in previous modeling studies of pure HCOOH ice irradiated at 15 K with 267 MeV Fe ions, conducted under a similar methodological approach of Moraes *et al.*^13^ The initial molecular composition of ice plays a determining role in radiation-induced chemical evolution. For example, in pure formic acid ice^13^ irradiated at 15 K by 267 MeV Fe ions, the authors have demonstrated that the main products formed are CO, CO_2_, and H_2_O, with HCOOH being rapidly consumed and acting predominantly as a transient source of radicals, rather than an efficient precursor of more complex organic molecules. In contrast, in the binary mixture H_2_O : HCOOH (1 : 1) ice irradiated under similar conditions, a more diverse chemical network is observed, with the additional formation of species such as CH_3_OH, H_2_CO, OH, and HCO, in addition to CO and CO_2_. This difference stems mainly from the presence of water, which introduces a chemistry rich in OH radicals and favors more efficient hydrogen abstraction and radical recombination mechanisms.

In the case of pure HCOOH ice, the dominant mechanisms are hydrogen abstraction and radical recombination, involving intermediates such as HOCO and OH, which mainly leads to fragmentation and the formation of simple, stable molecules. On the other hand, in matrices containing H_2_O, the availability of hydrogenated species and free radicals promotes additional reaction pathways, allowing the synthesis of partially hydrogenated molecules, such as methanol and formaldehyde, which are not produced significantly in pure ice. More importantly, the inclusion of water makes the experimental system significantly more representative of real astrophysical environments. In regions such as grain mantles in dense molecular clouds, protoplanetary disks, and the surfaces of icy bodies, water is typically the dominant component of the ice matrix. Thus, while pure HCOOH ice provides a simplified and useful view of the fundamental mechanisms of molecular fragmentation, it does not adequately capture the chemical complexity of these natural environments. In summary, while pure HCOOH ices tend to evolve into simple products dominated by CO and CO_2_, systems containing H_2_O favor a more complex and diverse chemistry and, crucially, more faithfully represent real astrophysical scenarios. Thus, the present study with H_2_O : HCOOH ice offers a more robust and physically relevant description of radiation-induced chemistry in interstellar ices than approaches based exclusively on pure HCOOH.

Table 3 presents the average parameters of the effective reaction rate constants (ERCs) and desorption yields for different irradiated ice systems under distinct experimental conditions. In general, experiments employing high-energy heavy ions exhibit higher ERC values for dissociation than those observed in electron-irradiated systems, reflecting the greater local energy deposition associated with the high braking power of these projectiles. This behavior is clearly observed when comparing HCOOH + H_2_O ice irradiated by Ni ions (9.1 × 10^−3^ s^−1^) with pure H_2_O ice irradiated by electrons (9.6 × 10^−2^ s^−1^), highlighting differences in interaction mechanisms and energy distribution. Desorption yields follow a similar trend, with heavy ions inducing significantly more efficient ejections (6.1 × 10^5^ molecules per ion in the present work), in contrast to electrons (3.2 × 10^−4^ molecules per electron), which reflects the higher ionization density along the heavy ion track.

**Table 3:** Comparison of the average values of the effective rate coefficients (ERCs) and desorption parameters for HCOOH in mixture with H_2_O and in pure form, obtained in the present model, with data from previous simulations based on the PROCODA code and results reported in the literature for different astrophysical ices

Average parameter	HCOOH + H_2_O at 15 K irradiated by 46 MeV ^58^Ni^11+^ (this work)	N_2_ : H_2_O ice at 15 K irradiated by 40 MeV Ni^11+^ (Queiroz *et al.* 2025)^23^	HCOOH ice at 15 K irradiated by 267 MeV ^56^Fe^22+^ (Moraes *et al.* 2026)^13^	H_2_O : O_2_ ice at 9 K irradiated by 0.8 MeV H^+^ (Silva *et al.* 2025)^22^	Pure H_2_O ice at 20 K irradiated by 2 keV electrons (Da Silveira & Pilling 2024)^20^
ERC for radiation- induced dissociation	9.1 × 10^−3^ s ^−1^	3.7 × 10^−3^ s^−1^	3.8 × 10^−1^ s^−1^	3 × 10^−3^ s^−1^	9.6 × 10^−2^ s^−1^
ERC for bimolecular collisions	1.2 × 10^−24^ cm^3^ molecules per s	2.7 × 10^−25^ s^−1^ cm (ref. 3) molecules per s	2.3 × 10^−23^ cm^3^ molecules per s	5.3 × 10^−25^ cm^3^ molecules per s	9.1 × 10^−24^ cm^3^ molecules per s
ERC for intrinsic desorption	3.68 × 10^−4^ s^−1^	5.4 × 10^−4^ s^−1^	1.36 × 10^−3^ s^−1^	8.3 × 10^−9^ s^−1^	1.2 × 10^−6^ s^−1^
Desorption rate	6.7 × 10^14^ molecules per s	6.4 × 10^13^ molecules per s	8.2 × 10^13^ molecules per s ^−1^	1.3 × 10^11^ molecules per s	9.5*e* + 10 molecules per s
Desorption yield	6.1 × 10^5^ molecules per ion	1.2 × 10^5^ molecules per ion	1.5 × 10^5^ molecules per ion	2.1 × 10^−1^ molecules per ion	3.2 × 10^−4^ molecules per electron

Focusing specifically on the comparison between the H_2_O : HCOOH ice (this work) and pure HCOOH ice, it is observed that the mixed system irradiated by heavy ions (46 MeV ^58^Ni^11+^, 15 K) presents an ERC value for dissociation of 9.1 × 10^−3^ s^−1^, while the pure HCOOH ice irradiated by 267 MeV ^56^Fe^22+^ exhibits a significantly higher value of 3.8 × 10^−1^ s^−1^. This difference of approximately two orders of magnitude indicates that the presence of H_2_O in the matrix acts as a moderator of HCOOH dissociation, possibly due to energy redistribution and the formation of a hydrogen bond network that dissipates the deposited energy. The presence of H_2_O substantially modifies the chemical dynamics of the system. Although the rate of direct dissociation is lower, mixed ice favors the formation of secondary radicals, especially OH, which promote additional reaction pathways and indirect consumption of HCOOH. Therefore, in the HCOOH + H_2_O system, the overall chemical processing tends to be more complex and efficient in generating new products, even if the primary destruction of the precursor molecule occurs at a lower rate.

Ice containing H_2_O also exhibits greater efficiency in intrinsic desorption processes (3.7 × 10^−4^ s^−1^) compared to pure HCOOH (1.36 × 10^−3^ s^−1^), as well as a higher overall desorption rate (6.7 × 10^14^ molecules per s *versus* 8.2 × 10^13^ molecules per s). These results indicate that the presence of water favors energy redistribution and the mobility of reactive species, promoting more efficient ejection of products from the solid matrix. Although the yield per ion is of the same order of magnitude (6.1 × 10^5^ for mixed ice and 1.5 × 10^5^ molecules ion^−1^ for pure ice), the system containing H_2_O exhibits greater overall efficiency due to its more complex chemical dynamics. These differences reflect the fundamental role of water as a moderator of radiation-induced chemistry. While pure HCOOH ice favors rapid and direct dissociation, resulting predominantly in simple fragments, mixed ice significantly broadens the diversity of chemical processes, including OH-mediated reactions and more efficient recombination mechanisms.

Table 4 below compares the values of the effective rate coefficients (ERCs) obtained using the PROCODA code for pure HCOOH ice and for HCOOH ice mixed with H_2_O. This comparison allows us to assess the influence of the aqueous matrix on the dominant reaction pathways, as well as on the efficiency of molecular formation and destruction processes in irradiated ice.

**Table 4:** Comparison of effective rate coefficients (ERCs) obtained from PROCODA calculations for pure HCOOH ice (obtained from ^13^) and H_2_O : HCOOH (1 : 1) ice (this work)

Reaction	Pure HCOOH ice (moraes *et al.* 2026)^13^	H_2_O : HCOOH (1 : 1) ice (this work)
H + C_2_H_3_ → CH_2_CH_2_	k24 = 4.23 × 10^−23^	k24 = 2.8 × 10^−23^
	cm^3^ molecules per s	cm^3^ molecules per s
CH + R → H + C	k13 = 2.9 × 10^−3^ s^−1^	k13 = 5.8 × 10^−3^ s^−1^
C + C_2_O_2_ → O_2_ + C_3_	k197 = 7.4 × 10^−26^	k197 = 1.5 × 10^−24^
	cm^3^ molecules per s	cm^3^ molecules per s
CH_4_ + R → H + CH_3_	k19 = 6.0 × 10^−1^ s^−1^	k19 = 6.3 × 10^−3^ s^−1^
O + H_2_O_2_ → H_2_ + O_3_	k135 = 1.3 × 10^−24^	k135 = 2.5 × 10^−24^
	cm^3^ molecules per s	cm^3^ molecules per s
HO_2_ + R → O + OH	k110 = 9.7 × 10^−4^ s^−1^	k110 = 4.4 × 10^−3^ s^−1^

In general terms, it is observed that the ERCs vary by several orders of magnitude depending on the chemical environment, reflecting the strong dependence of the effective rates on the ice matrix and the associated energy transfer processes. More specifically, bimolecular reactions involving radicals show extremely low values in pure ice, as in the case of C + C_2_O_2_ → O_2_ + C_3_ (∼7.4 × 10^−26^ s^−1^), while in the mixture with water there is a significant increase (∼1.5 × 10^−24^ s^−1^), indicating that the presence of H_2_O favors radical mobility and recombination. An even more striking behavior is observed for H + C_2_H_3_ → CH_2_CH_2_, whose ERC increases from ∼4.23 × 10^−23^ s^−1^ (pure) to ∼2.8 × 10^−3^ s^−1^ (mixture), demonstrating that the aqueous matrix acts as an effective catalytic medium for radical reactions.

On the other hand, direct dissociation processes show distinct trends. The reaction CH_4_ + R → H + CH_3_ shows a significant reduction in the ERC when transitioning from pure ice (∼6.0 × 10^−1^ s^−1^) to the mixture (∼6.3 × 10^−3^ s^−1^), suggesting that the water-rich environment may stabilize precursor species. In contrast, other dissociation reactions, such as HO_2_ + R → O + OH, show a moderate increase (from ∼9.7 × 10^−4^ to ∼4.4 × 10^−3^ s^−1^), indicating that the effect of the medium depends on the balance between molecular stabilization and facilitation of reactive channels. These results reinforce that the ERCs obtained by PROCODA are not merely adjusted kinetic parameters, but reflect emergent physicochemical properties of the irradiated system, such as diffusion, radical trapping, and energy redistribution in the ice matrix. Consequently, such coefficients are fundamental for reproducing the temporal evolution of molecular abundances and explaining the rapid approach to chemical equilibrium (∼2000 s), as well as providing quantitative support for the interpretation of astronomical observations of cold environments, such as star-forming regions.

## 4 Astrophysical implications

The presence of molecular ices in cold astrophysical environments, such as dense molecular clouds, protoplanetary disks and surfaces of outer Solar System objects, plays a central role in the chemistry of the interstellar medium. In these regions, volatile compounds such as H_2_O, CO, CO_2_ and HCOOH are incorporated into dust grains at temperatures of around 10 K, where they are subjected to continuous ionizing radiation, mainly UV photons, electrons, protons and galactic heavy ions, over time scales ranging from thousands to millions of years.^42,43^ Given the impossibility of directly reproducing such time scales in the laboratory, the experimental approach is based on the application of intensified radiation fluxes to accelerate the physicochemical processes. This methodology is complemented by kinetic computational codes, such as PROCODA, which allow extrapolating laboratory results to real astrophysical conditions. PROCODA uses coupled reaction networks, parameterized by effective rate constants derived from experimental data, to simulate the temporal evolution of chemical compositions in irradiated ices.

By integrating laboratory-derived spectroscopic data with computational physicochemical modeling, it is possible to infer the formation, destruction, and stability of complex molecules under cosmic conditions. This hybrid approach allows estimating, for example, the survival time of molecular species, the degree of radiation-induced desorption, and the viability of prebiotic pathways on icy surfaces exposed to interstellar radiation. Thus, the study of the evolution of ices such as HCOOH mixed with H_2_O becomes a representative model for understanding the processes of molecular complexification in astrophysical environments.^44,45^ The PROCODA model captures the cumulative chemical effects of ionization by high-LET agents, particularly heavy ions. Although complementary processes such as photolysis and electron irradiation occur in space, their inclusion requires dedicated treatment beyond the scope of the current study.

Astrochemistry experiments aim to replicate the harsh conditions of the interstellar medium, where molecules are frozen onto dust grains and irradiated over millions of years. To simulate this, laboratories employ ultra-high vacuum chambers and cryogenic systems operating at 10–50 K, temperatures typical of dense molecular clouds and icy solar system bodies. The equivalence between laboratory and cosmic irradiation times is determined by the ratio of particle fluxes, following the relation: *t*_e_ = (*ϕ*_lab_/*ϕ*_e_) × *t*_lab_, where *t*_e_ is the equivalent cosmic timescale, *ϕ*_lab_ and *ϕ*_e_ are the particle fluxes in the laboratory and in space, respectively, and *t*_lab_ is the duration of laboratory irradiation.

Computational astrochemical models are fundamental for studying the molecular evolution of ices under space irradiation, involving processes like desorption, dissociation, and solid-phase reactions. Codes such as NAUTILUS, MONACO, and ProDiMo simulate gas-phase and surface chemistry but often treat the effects of ionizing radiation on ices in a simplified manner. In contrast, the PROCODA model represents a significant advance by using effective rate coefficients derived from laboratory data to solve systems of coupled differential equations. This enables the extrapolation of short-duration experiments to simulate chemical evolution over millions of years in space.

PROCODA has successfully reproduced distinct chemical pathways and equilibrium times for CO_2_ ices under various radiation types (ions, UV, electrons), without requiring explicit knowledge of the projectile energy, but rather using integrated chemical effects. In this work, the model is applied to H_2_O : HCOOH ice irradiated by heavy ions, enabling the simulation of galactic cosmic ray processing and predicting the evolution of species such as CO, CO_2_, H_2_CO, and H_2_O until chemical equilibrium phase (*i.e.*, a radiation-induced dynamic steady state) is reached. By calibrating differential equations with experimental spectroscopic data, PROCODA provides reliable estimates of molecular abundances in irradiated astrophysical ices, especially ices toward protostars, values that can be directly compared with astronomical observations. Instruments like the James Webb Space Telescope (JWST), with detection limits around 10^16^ molecules cm^−2^, have revolutionized infrared spectroscopy of ices, surpassing the sensitivity of the Infrared Space Observatory (ISO) and allowing detection of both major and minor species, including H_2_O, CO_2_, CH_3_OH, and H_2_CO. PROCODA thus serves as a robust tool for bridging laboratory simulations and observational astrochemistry.

**Fig. 5:**
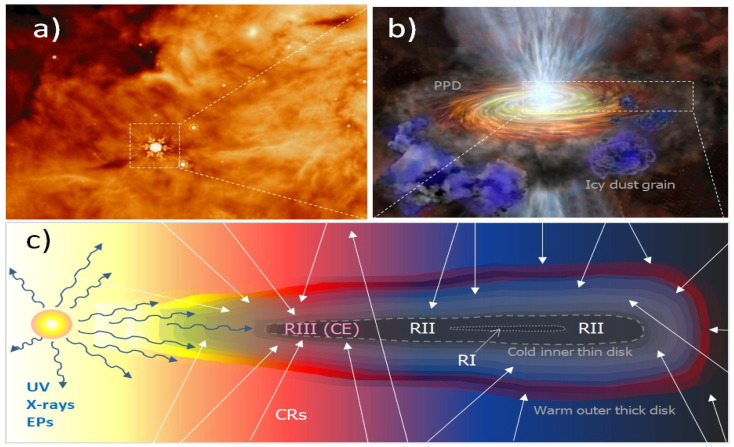
Illustration of irradiation-driven processing in a protoplanetary disk. Panel (a) shows a real infrared image of glowing clouds of gas and dust surrounding the protostar IRAS 2A (source: https://science.nasa.gov). Panel (b) presents an artist's rendition of a protostar with its surrounding protoplanetary disk (PPD), highlighting icy dust grains exposed to energetic radiation. Panel (c) illustrates the conceptual model of the irradiated disk structure, indicating the penetration of various ionizing agents—UV photons, X-rays, and energetic particles (EPs)—as well as cosmic rays (CRs). Different disk regions (RI, RII, RIII) are labeled, with RIII (CE) denoting the inner region where chemical equilibrium is expected to occur first due to prolonged irradiation and molecular reprocessing. Cold and warm disk layers are also indicated.

Fig. 5 illustrates the irradiation-driven chemical processing occurring in protostellar environments, with a focus on the disk surrounding the protostar IRAS 2A. Panel (a) displays an infrared image of glowing gas and dust in the outer envelope of the protostar, as observed by NASA, while panel (b) presents an artist's impression of a protostellar system with a surrounding protoplanetary disk (PPD), showing icy dust grains embedded within the disk structure. Panel (c) conceptualizes the spatial distribution of radiative and energetic particle fluxes within the protostellar environment and highlights three chemically distinct regions, based on their exposure to stellar and cosmic irradiation. This figure illustrates three chemically distinct regions in protostellar disks. Region I (RI) includes the outermost, coldest layers where shielding from stellar radiation is strongest. Here, low temperatures (∼15 K) favor ice preservation, and chemical changes are primarily driven by galactic cosmic rays. Region II (RII) is an intermediate zone exposed to moderate stellar radiation (UV and X-rays), enhancing photochemistry and partial desorption, especially of more refractory ices. Region III (RIII), lies closest to the protostar and is expected to be the first within the chemical equilibrium (CE) phase, is dominated by intense radiation and energetic particles. In this dynamic region, formation and destruction rates tend toward equilibrium, though ices may persist in shielded dust grain niches. These stratified regions help contextualize PROCODA's simulations within realistic astrophysical settings.

To assess the astrophysical validity of the molecular abundances predicted by the PROCODA model, we performed a comparative analysis using spectral data from the Infrared Space Observatory (ISO) for the protostar W33A.^46^ The model simulation considered a H_2_O : HCOOH (1 : 1) ice mixture irradiated at 15 K, with molecular abundances computed at the CE phase. Absolute column densities (in molecules cm^−2^) were calculated for 73 relevant species using the PROCODA code, reflecting the composition expected in Region III (RIII) of the protostellar disk, where irradiation-driven equilibrium dominates, as illustrated in Fig. 5.

**Fig. 6:**
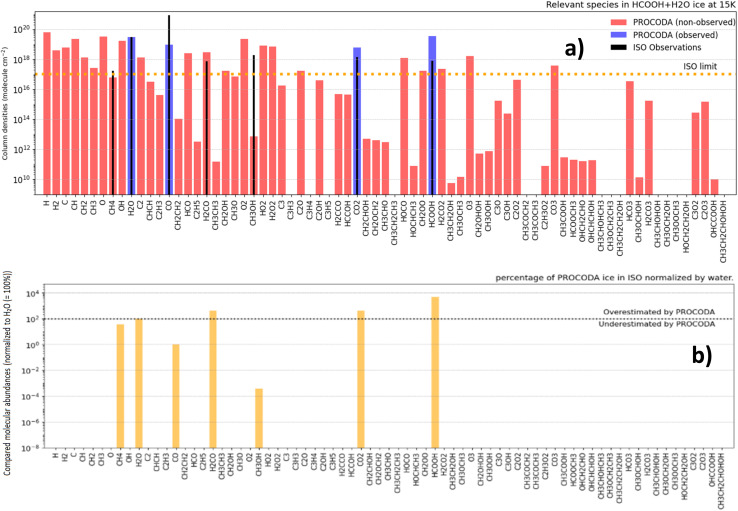
Comparison between observed and modeled molecular abundances for ices toward the protostar W33A and those simulated by PROCODA for irradiated H_2_O : HCOOH (1 : 1) ice at 15 K, at chemical equilibrium. Panel (a) shows absolute column densities normalized to the observed H_2_O abundance. Red bars indicate species predicted by PROCODA but not detected; blue bars represent species both predicted and observed; black bars correspond to ISO observations. The orange dashed line marks the ISO detection limit (∼10^17^ molecules cm^−2^). Panel (b) presents the relative agreement (in %) between PROCODA and ISO abundances, normalized to H_2_O. The black dotted line at 100% indicates perfect agreement; values above or below reflect model overestimation or underestimation, respectively.

Fig. 6a presents a direct comparison between the column densities predicted by PROCODA (red and blue bars) and the molecular species effectively detected in W33A by ISO (black bars). Red bars denote species predicted by PROCODA that have not been observed, while blue bars represent model predictions for species also identified in the ISO spectrum. All PROCODA values were normalized to the H_2_O column density observed by ISO telescope for W33A. The horizontal yellow dashed line indicates ISO's typical detection threshold (∼10^17^ molecules cm^−2^), enabling a clear visual distinction between molecules whose abundances are detectable and those that fall below the sensitivity limit.

The agreement between model and observation is particularly evident for species such as H_2_O, CH4, H_2_CO, and CO, which are all predicted by PROCODA with abundances consistent with ISO measurements. This correspondence reinforces the model's ability to simulate realistic chemical outcomes under astrophysical conditions. Conversely, many predicted species lie below the detection limit, either because they are present at trace levels or because their vibrational modes produce weak infrared absorption features not easily resolved in ISO data. Nonetheless, these results highlight the utility of PROCODA not only for matching observational data but also for forecasting the existence of undetected, yet chemically plausible, species, many of which may become observable with the increased sensitivity of next-generation instruments such as JWST.

Fig. 6b presents the relative agreement (in %) between PROCODA and ISO abundances, normalized to H_2_O. The yellow bars display the ratio between the PROCODA-predicted and ISO-observed values for each molecular species in the W33A protostar. The dotted black line at 100% serves as a reference for perfect agreement. Species above the 100% threshold are overestimated by the model, while those below are underestimated. This graphical approach allows a clearer assessment of model accuracy, independent of the absolute values. The analysis shows that the key ice constituents detected by ISO (*e.g.* H_2_O, CO_2_, and HCOOH) are reasonably well reproduced by the PROCODA model in terms of relative abundance, reinforcing its validity. Discrepancies observed for some species may arise from differences in experimental and astrophysical conditions, such as ice composition, irradiation dose, and temperature, as well as from the limited infrared sensitivity of ISO telescope for certain molecules. Certain species, such as CH_3_OH and CO, are significantly underestimated by the current modeling approach. This discrepancy suggests that additional ice components or alternative irradiation scenarios may be required to fully account for their observed abundances in W33A.

Overall, Fig. 6b highlights the strength of the PROCODA approach in reproducing observed abundance trends while also identifying species whose detection may require more sensitive instruments like JWST. The consistency in relative abundances further supports the use of PROCODA for extrapolating laboratory-based simulations to realistic astrochemical environments.

The inclusion of H_2_O in the initial ice matrix leads to a substantially improved agreement with astronomical observations compared to previous models that considered only pure HCOOH irradiated ices *e.g.* Moraes *et al.*^13^ This improvement is physically consistent with the well-established dominance of water as the primary constituent of interstellar and circumstellar ices, where it acts not only as a passive matrix but also as an active chemical environment. The presence of H_2_O modifies the physicochemical properties of the ice, including its dielectric constant, hydrogen-bonding network, and energy dissipation pathways, which in turn influence the radiolytic processing induced by energetic ions. As a result, reaction rates, branching ratios, and molecular stabilization pathways differ significantly from those observed in pure HCOOH systems.

From a microscopic perspective, the incorporation of H_2_O enhances the formation and stabilization of reactive intermediates such as OH, H, and secondary electrons, which play a central role in driving complex chemistry under irradiation. These species can efficiently interact with HCOOH and its dissociation fragments including C-bearing species, promoting secondary reaction channels that are absent or strongly suppressed in pure ice simulations mainly in the beginning of irradiation. Consequently, the chemical network becomes more representative of realistic astrophysical environments, leading to product distributions and column densities that better reproduce observational constraints derived from infrared and radioastronomical data.

Moreover, the inclusion of water ice introduces important structural and dynamical effects, such as porosity, trapping efficiency, and diffusion barriers, which critically impact the retention and mobility of newly formed species. These factors are essential for accurately modeling the evolution of molecular abundances over astrophysical timescales. In contrast, models based solely on pure HCOOH ices neglect these matrix effects, resulting in systematic deviations from observed abundances and spectral features. Therefore, the present model, by incorporating H_2_O as a major component of the ice matrix, provides a more realistic and robust framework for interpreting astrochemical processes in irradiated icy environments, thereby achieving a closer agreement with astronomical observations.

To further evaluate the accuracy of the PROCODA model in predicting molecular abundances in astrophysical ices, a second comparative analysis was conducted with recent observational data from the James Webb Space Telescope (JWST) for the protostar IRAS 2A (*e.g.* ref. 47 and 48). These data provide high-sensitivity infrared spectra of cold molecular ices in the protostellar environment, allowing a more refined comparison with computational simulations.

**Fig. 7:**
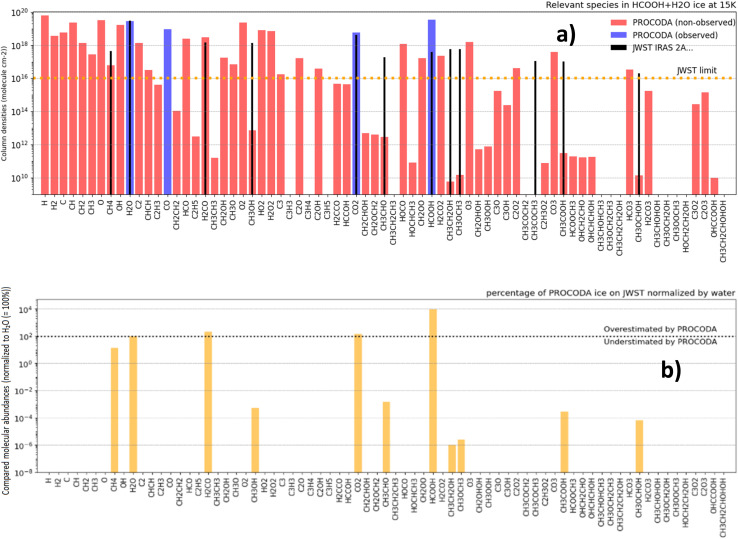
Comparison between observed and modeled molecular abundances for ices toward the protostar IRAS 2A and those simulated by PROCODA for irradiated H_2_O : HCOOH (1 : 1) ice at 15 K, at chemical equilibrium. Panel (a) shows absolute column densities normalized to the observed H_2_O abundance. Red bars indicate species predicted by PROCODA but not detected; blue bars represent species both predicted and observed; black bars correspond to JWST observations. The orange dashed line marks the JWST detection limit (∼10^16^ molecules cm^−2^). Panel (b) presents the relative agreement (in %) between PROCODA and JWST abundances, normalized to H_2_O. The black dotted line at 100% indicates perfect agreement; values above or below reflect model overestimation or underestimation, respectively.

Fig. 7a presents the absolute column densities (in molecules cm^−2^) of species predicted by PROCODA for H_2_O : HCOOH (1 : 1) ice irradiated at 15 K, at the chemical equilibrium phase, and compares them with the column densities observed by JWST. Red bars indicate species predicted by the model but not yet observed, while blue bars represent model predictions that are supported by JWST detections. Black bars correspond to the observational data for IRAS 2A. All model results were normalized to the observed H_2_O abundance to allow consistent comparison. The horizontal dashed orange line (∼10^16^ molecules cm^−2^) indicates the typical JWST detection threshold. The plot highlights several species, such as H_2_O, H_2_CO, and CO_2_, which are both predicted by PROCODA and observed in the JWST spectra with strong agreement in magnitude, supporting the robustness of the model. Additionally, several species predicted by PROCODA fall below the JWST detection limit, suggesting either low abundance or limited infrared activity.

Once more, to quantify the degree of agreement between model and observation, Fig. 7b provides a relative comparison of molecular abundances, normalized to H_2_O (=100%), which is a standard approach in observational astrochemistry. The yellow bars represent the percentage ratio of PROCODA-predicted to JWST-observed abundances. The dotted black line marks the 100% reference point, denoting perfect agreement. Values above the line indicate species overestimated by the model, while those below represent underestimations. Once more, certain species such as CH_3_OH and other larger organic molecules are significantly underestimated by the current modeling approach. Conversely, the abundance of formic acid seems to be overestimated under the same conditions. These discrepancies suggest that more complex ice mixtures or alternative irradiation scenarios may be necessary to accurately reproduce the molecular abundances observed also in this protostar. Comparison also reveals potentially important molecules that have not yet been detected but are consistently predicted by the model, such as CH_2_OH and HOCH_2_CHO, underscoring the model's capacity for chemical forecasting.

In general, the agreement between molecular abundances predicted by the PROCODA model and those observed toward protostars such as IRAS 2A and W33A reinforces the model's reliability and astrophysical relevance. For IRAS 2A, recent JWST data report relative abundances of ∼25–30% CO_2_, ∼5–10% CH_3_OH, and ∼1–3% HCOOH with respect to H_2_O (*e.g.* ref. 47 and 48) which are in close agreement with PROCODA simulations of H_2_O : HCOOH (1 : 1) ices irradiated by heavy ions at 15 K. Similarly, for W33A (see also ref. 46, 49 and 50), indicate up to 15–20% CO_2_, ∼5% CH_3_OH, and ∼3–4% HCOOH, values that are also well reproduced by the model. These correlations depend on the compatibility between laboratory and astrophysical conditions, including radiation type, ice composition, and temperature. Spectral limitations must also be considered, as some PROCODA-predicted species may fall below instrumental detection thresholds or lie outside the spectral range. Nevertheless, PROCODA stands out as a robust tool for interpreting astronomical spectra, facilitating the identification of molecular bands and predicting chemically plausible species yet to be detected.

These results underscore the significance of the PROCODA model as a powerful tool for simulating the chemical evolution of irradiated astrophysical ices, particularly in protoplanetary disks and other cold space environments exposed to ionizing radiation. By employing experimental-based column densities data and solving coupled differential equations providing numerical values hundreds effective rate coefficients, PROCODA provides accurate predictions of molecular abundances that align well with state-of-the-art astronomical observations. Furthermore, the methodology of comparing simulated and observed spectra, normalized by H_2_O and analyzed across detection thresholds, offers a systematic framework for assessing model validity and guiding future observational campaigns. Such predictive capabilities are essential for anticipating molecular signatures in upcoming missions and for deepening our understanding of the chemical inventory in star-forming regions, icy bodies, and interstellar clouds.

A central aspect is the role of radiation-induced desorption, which promotes the transition of chemical species from ice to the gas phase. This process ensures that molecules formed or transformed in ice, such as formic acid, can be effectively detected by high-sensitivity radio telescopes, such as ALMA, in protoplanetary disks and other young systems. Thus, the results of this work not only contribute to understanding the mechanisms of retention and release of volatile species but also provide predictive support for astronomical detection campaigns in the millimeter and submillimeter domains, consolidating the bridge between laboratory/computational models and observations. Although the detection of HCOOH in protoplanetary disks is still unique, the compound has been observed in star-forming environments, such as protostellar shocks, for example, in L1157-B1, which reinforces the importance of desorption mechanisms in making these molecules available in the gas.^51^ Furthermore, UV photodesorption studies on ices containing HCOOH show how relevant and sensitive this process is to explain its presence in the gas phase.

Finally, it is worth noting that although the present H_2_O : HCOOH ice analogue provides valuable insights into the radiation-induced chemistry of astrophysical ices, it represents only a simplified approximation of the much more complex composition of interstellar and protostellar ice mantles. Real astrophysical ices may contain significant abundances of CO, CO_2_, CH_3_OH, NH_3_, CH_4_, ionic species, and salts, while their chemical evolution is also influenced by thermal processing and exposure to multiple energetic agents, including UV photons, X-rays, cosmic rays, and energetic particles. Therefore, the results presented here should be interpreted within the context of the specific ice composition and irradiation conditions investigated, while future studies involving more complex ice mixtures and processing scenarios will be required to achieve a more comprehensive description of astrophysical ice chemistry.

## 5 Conclusion

This study presents a detailed computational investigation of radiolytic chemistry and desorption dynamics in astrophysical ices composed of H_2_O : HCOOH (1 : 1), irradiated with high-energy Ni ions at 15 K. Leveraging the PROCODA model, calibrated against experimental data from Bergantini *et al.*,^10^ we simulate the time-resolved chemical evolution and molecular desorption from the solid phase, with the goal of reproducing and interpreting key features observed in astronomical environments. The principal findings are outlined below:

(1) Rapid chemical evolution toward equilibrium: the irradiated ice mixture reaches a chemically stable regime (chemical equilibrium) within approximately 1000 seconds of irradiation, characterized by constant column densities of major species.

(2) Identification of dominant products: key molecules formed in the simulation include CO, CO_2_, CH_3_OH, H_2_CO, OH, and HCO, species commonly observed in interstellar and circumstellar environments. Their abundances and temporal behaviors are consistent with both laboratory data and astrophysical spectra.

(3) Dynamic desorption behavior: the model predicts time-dependent desorption rates for volatile and semi-volatile species, with transitory peaks for certain molecules, indicating that radiation-induced desorption is not uniform over time. This behavior suggests distinct desorption regimes in early and late stages of irradiation.

(4) Comparison with astronomical observations: the simulated molecular abundances at chemical equilibrium show good agreement with some frozen species observed by ISO telescope toward the protostar W33A (*e.g.* H_2_O, CH_4_, H_2_CO, and CO_2_). In the case of JSWT observations of the protostar IRAS 2A, we obtain a very good match with PROCODA model for the species H_2_O, CO_2_ and H_2_CO. This comparison underscores the capability of the PROCODA code to serve as an accurate predictive instrument in astrochemical modeling.

(5) Prediction of non-observed but chemically viable species: several species predicted by PROCODA fall below the ISO and JWST detection threshold but may be within the detection limits of future telescopes, offering targets for future observations.

(6) Astrochemical implications for protoplanetary disks: the results confirm that radiation-driven chemistry in cold, shielded regions of protostellar disks can lead to the formation and desorption of prebiotic molecules, reinforcing the role of cosmic rays in shaping molecular complexity in space.

(7) The inclusion of H_2_O in the ice matrix provides a more realistic representation of interstellar ices than pure HCOOH models Moraes *et al.*,^13^ as water modifies radiolytic chemistry *via* hydrogen bonding, energy dissipation, and the production of reactive species such as OH and H. Consequently, mixed H_2_O : HCOOH ices yield chemical pathways and desorption behaviors more consistent with astronomical observations, supporting the need for water-rich astrochemical models.

A key contribution of this study lies in the numerical quantification chemical equilibrium abundance and of desorption for the analyzed species, offering direct parameters to enhance the interpretation of radio telescope observations. By characterizing the relative intensities and desorption regimes, the model enables a more accurate translation of interstellar spectral signatures into true molecular abundances, thereby reducing uncertainties in emission line models. These insights not only support the identification of molecules such as formic acid, already observed in the gaseous phase within star-forming regions, but also guide the selection of promising targets for high-sensitivity instruments like ALMA. Overall, the results underscore the value of the PROCODA model in bridging laboratory astrochemistry with astronomical data, paving the way for future applications that could deepen our understanding of molecular evolution in the early stages of planetary system formation, particularly through observations from next-generation facilities such as JWST and ALMA.

## Conflicts of interest

The authors declare that they have no known competing financial interests or personal relationships that could have appeared to influence the work reported in this paper.

## Appendix A1

**Table 5:** Modeled numerical values obtained from the best-fit model for radiolysis-induced desorption at the chemical equilibrium phase in the studied H_2_O : HCOOH ice irradiated by heavy ions

Species	Desorption in column densities (molecules cm^−2^)	Desorption in percentage (%)
H	2.5 × 10^18^	7.7 × 10^1^%
H_2_	1.4 × 10^15^	4.3 × 10^−2^%
C	4.1 × 10^15^	1.3 × 10^−1^%
CH	1.6 × 10^16^	4.9 × 10^−1^%
CH_2_	3.1 × 10^14^	9.4 × 10^−3^%
CH_3_	3.2 × 10^13^	9.7 × 10^−4^%
O	3.8 × 10^16^	1.1 × 10^0^%
CH_4_	1.4 × 10^12^	4.1 × 10^−5^%
OH	2.6 × 10^16^	8.0 × 10^−1^%
H_2_O	3.9 × 10^17^	1.2 × 10^1^%
C_2_	7.5 × 10^14^	2.2 × 10^−2^%
CHCH	1.5 × 10^11^	4.5 × 10^−5^%
CO	8.7 × 10^15^	2.6 × 10^−1^%
HCO	3.2 × 10^14^	9.6 × 10^−3^%
H_2_CO	5.1 × 10^15^	1.5 × 10^−1^%
CH_2_OH	4.6 × 10^12^	1.3 × 10^−4^%
CH_3_O	1.5 × 10^12^	4.5 × 10^−5^%
O_2_	1.0 × 10^17^	2.9 × 10^0^%
HO_2_	2.4 × 10^15^	7.3 × 10^−2^%
H_2_O_2_	2.0 × 10^16^	6.1 × 10^−1^%
C_3_	3.1 × 10^10^	9.2 × 10^−5^%
C_2_O	6.4 × 10^12^	1.9 × 10^−4^%
C_2_OH	7.7 × 10^10^	2.3 × 10^−6^%
CO_2_	5.4 × 10^15^	1.6 × 10^−1^%
HOCO	8.0 × 10^13^	2.3 × 10^−3^%
CH_2_OO	6.3 × 10^11^	1.9 × 10^−5^%
HCOOH	1.4 × 10^17^	4.3 × 10^0^%
H_2_CO_2_	1.8 × 10^12^	5.6 × 10^−5^%
O_3_	2.0 × 10^14^	6.1 × 10^−3^%
C_2_O_2_	2.0 × 10^11^	6.2 × 10^−5^%
CO_3_	7.1 × 10^12^	2.2 × 10^−4^%
HCO_3_	2.2 × 10^11^	6.3 × 10^−6^%

**Table 6:** Numerical values for the modeled abundances at the chemical equilibrium phase in the studied H_2_O : HCOOH ice irradiated by heavy ions

Specie	Abundances (molecules cm^−2^)	Abundances (%)
H	3.7 × 10^18^	2.2 × 10^1^%
H_2_	2.2 × 10^17^	1.3 × 10^0^%
C	3.5 × 10^17^	2.2 × 10^0^%
CH	1.3 ×10^18^	8.3 × 10^0^%
CH_2_	7.8 × 10^16^	4.8 × 10^−2^%
CH_3_	1.6 × 10^16^	9.9 × 10^−2^%
O	1.9 × 10^18^	1.1 × 10^1^%
CH_4_	3.6 × 10^15^	2.2 × 10^−3^%
OH	9.9 × 10^17^	6.9 × 10^1^%
H_2_O	1.7 × 10^18^	1.1 × 10^1^%
C_2_	7.8 × 10^16^	4.8 × 10^−1^%
CHCH	1.9 × 10^15^	1.2 × 10^−3^%
C_2_H_3_	2.3 × 10^14^	1.0 × 10^−4^%
CO	5.3 × 10^17^	3.3 × 10^0^%
CH_2_CH_2_	6.2 × 10^12^	3.3 × 10^−5^%
HCO	1.4 × 10^17^	9.4 × 10^0^%
C_2_H_5_	1.8 × 10^11^	9.1 × 10^−5^%
H_2_CO	1.7 × 10^17^	1.1 × 10^−1^%
CH_3_CH_3_	9.9 × 10^9^	5.5 × 10^−8^%
CH_2_OH	1.0 × 10^16^	6.3 × 10^−3^%
CH_3_O	4.0 × 10^15^	2.5 × 10^−2^%
O_2_	1.3 × 10^18^	8.5 × 10^1^%
CH_3_OH	4.3 × 10^11^	2.6 × 10^−6^%
HO_2_	4.8 × 10^17^	2.9 × 10^−1^%
H_2_O_2_	4.0 × 10^17^	2.5 × 10^−1^%
C_3_	1.0 × 10^15^	6.0 × 10^−3^%
C_2_O	9.9 × 10^15^	6.1 × 10^−2^%
C_2_OH	2.2 × 10^15^	1.4 × 10^−4^%
H_2_CCO	2.7 × 10^14^	2.0 × 10^−4^%
HCCOH	2.6 × 10^14^	2.0 × 10^−3^%
CO_2_	3.5 × 10^17^	2.2 × 10^0^%
CH_2_CHOH	2.8 × 10^11^	1.8 × 10^−7^%
CH_2_OCH_2_	2.2 × 10^11^	1.4 × 10^−7^%
CH_3_CHO	1.7 × 10^11^	1.6 × 10^−7^%
HOCO	7.1 × 10^16^	4.4 × 10^−1^%
HOCHCH_3_	4.6 × 10^9^	2.8 × 10^−8^%
CH_2_OO	9.8 × 10^15^	6.1 × 10^−2^%
HCOOH	2.1 × 10^18^	1.3 × 10^1^%
H_2_CO_2_	1.3 × 10^16^	8.3 × 10^−2^%
CH_3_CH_2_OH	3.3 × 10^8^	2.5 × 10^−9^%
CH_3_OCH_3_	8.4 × 10^8^	5.1 × 10^−8^%
O_3_	9.2 × 10^16^	5.7 × 10^−1^%
CH_2_OHOH	2.9 × 10^10^	1.8 × 10^−7^%
CH_3_OOH	4.3 × 10^10^	6.2 × 10^−5^%
C_3_O	9.9 × 10^13^	1.0 × 10^−3^%
C_3_OH	1.3 × 10^13^	8.3 × 10^−4^%
C_2_O_2_	2.4 × 10^15^	1.5 × 10^−3^%
C_2_H_3_O_2_	4.4 × 10^9^	2.7 × 10^−8^%
CO_3_	2.2 × 10^16^	1.4 × 10^−1^%
CH_3_COOH	1.7 × 10^10^	1.1 × 10^−7^%
HCOOCH_3_	1.1 × 10^10^	7.1 × 10^−8^%
OHCH_2_CHO	9.7 × 10^9^	5.9 × 10^−8^%
OHCHCHOH	1.0 × 10^10^	6.4 × 10^−8^%
HCO_3_	2.0 × 10^15^	1.3 × 10^−2^%
CH_3_OCHOH	7.8 × 10^8^	4.8 × 10^−8^%
H_2_CO_3_	1.0 × 10^14^	6.1 × 10^−5^%
C_3_O_2_	1.6 × 10^13^	9.9 × 10^−4^%
C_2_O_3_	8.4 × 10^13^	5.1 × 10^−4^%
OHCCOOH	5.7 × 10^8^	3.5 × 10^−8^%

## Supplementary Material

RA-OLF-D6RA03525F-s001

## Data Availability

The data underlying this article will be shared on reasonable request to the corresponding author. Supplementary information (SI) can be obtained online at https://doi.org/10.5281/zenodo.18379529. See also the PROCODA online database at https://www.univap.br/procoda. Supplementary information is available. See DOI: https://doi.org/10.1039/d6ra03525f.
